# Phytochemical Profiling, Anti-Inflammatory Action, and Human Gut Microbiota-Assisted Digestion of *Rheum officinale* Petiole and Root Extracts—An In Vitro Study

**DOI:** 10.3390/nu17213455

**Published:** 2025-11-01

**Authors:** Oleksandra Liudvytska, Mariusz Kowalczyk, Justyna Krzyżanowska-Kowalczyk, Karolina Michaś, Maria Michalak, Aneta Balcerczyk, Weronika Skowrońska, Marcin Równicki, Agnieszka Bazylko, Monika A. Olszewska, Joanna Kolodziejczyk-Czepas

**Affiliations:** 1 Department of General Biochemistry, Faculty of Biology and Environmental Protection, University of Lodz, Pomorska 141/143, 90-236 Lodz, Poland; oleksandra.liudvytska@biol.uni.lodz.pl (O.L.); karolina.michas@edu.uni.lodz.pl (K.M.); maria.michalak.02@gmail.com (M.M.); 2 Department of Phytochemistry, Institute of Soil Science and Plant Cultivation, State Research Institute, Czartoryskich 8, 24-100 Puławy, Poland; mkowalczyk@iung.pulawy.pl (M.K.); jkrzyzanowska@iung.pulawy.pl (J.K.-K.); 3 Department of Oncobiology and Epigenetics, Faculty of Biology and Environmental Protection, University of Lodz, 90-236 Lodz, Poland; aneta.balcerczyk@biol.uni.lodz.pl; 4 Department of Pharmaceutical Biology, Faculty of Pharmacy, Medical University of Warsaw, Banacha 1, 02-097 Warsaw, Poland; weronika.skowronska@wum.edu.pl (W.S.); agnieszka.bazylko@wum.edu.pl (A.B.); 5 Microbiota Laboratory, Department of Pharmaceutical Microbiology and Bioanalysis, Medical University of Warsaw, 1 Banacha St., 02-097 Warsaw, Poland; marcin.rownicki@wum.edu.pl; 6 Department of Pharmacognosy, Faculty of Pharmacy, Medical University of Lodz, 1 Muszynskiego St., 90-151 Lodz, Poland; monika.olszewska@umed.lodz.pl

**Keywords:** anti-inflammatory, cyclooxygenase-2, 5-lipoxygenase, endothelial cells, *Rheum officinale*, *Rhei* Radix et Rhizoma, metabolite profiling, stilbenes, anthraquinones

## Abstract

**Background/Objectives:** *Rheum officinale*, an ethnomedicinal plant, has roots widely employed in modern pharmacological formulations. However, many of its biological activities remain only partly recognized. Furthermore, the metabolome and biological activity of its edible petioles, often considered a waste product, have received limited scientific attention. **Methods and Results:** The examination of anti-inflammatory properties of both root and petiole extracts (1–50 µg/mL) revealed the inhibition of the pro-inflammatory cytokine release from human peripheral blood mononuclear cells, a reduction in *ALOX5* gene expression in human umbilical vein endothelial cells, and the significant inhibition (>60%) of cyclooxygenase-2 and 5-lipoxygenase activities. Importantly, no cytotoxic effects were detected at the tested concentrations. **Conclusions:** The petiole extract demonstrated anti-inflammatory efficiency comparable to, or exceeding that of the root extract, suggesting that *R. officinale* petioles could be valuable source of bioactive compounds for future investigations.

## 1. Introduction

Rhubarb (*Rheum* L.) is currently gaining global recognition and is utilized for the production of medicinal materials, primarily derived from its underground parts. Among the best-known rhubarb species is *Rheum officinale* Baill. According to the regulations of the Chinese Pharmacopeia, *R. officinale* Baill., together with *R. palmatum* L. and *R. tanguticum* Maxim. ex Balf. (all belonging to the section *Palmata*), is recognized as the source plant of the commercial *Rhei* Radix et Rhizoma (known as Dahuang in Chinese), one of the oldest and most frequently used components of Traditional Chinese Medicine (TCM) [1,2,3]. *Rhei* Radix (rhubarb root) is also listed in the pharmacopeias of 19 other countries, including several in Europe [4,5].

Ethnomedicinal uses of *R. officinale* include the treatment of gastrointestinal disorders, fever, blood purification, detoxification, removing blood stasis, preventing chronic renal failure [6], cancer [7], and promoting menstruation [8]. Moreover, the root of rhubarb has applications in various industries. The most important is its medicinal value and application in the pharmaceutical sector for producing phytotherapeutics and functional supplements (that promote health and well-being). Several preparations, including capsules, drops, mouthwashes, topical preparations, and cosmetics available on the market, contain rhubarb root [9]. Finally, it is used to produce natural dyes for cosmetics, textiles (such as wool or silk), and food colorants [10].

Due to the ever-increasing market demand for *R. officinale* root and the environmental damage caused by excessive harvesting and overexploitation of natural sources, large-scale rhubarb cultivation, particularly in China, has emerged as a significant commercial practice [1,11,12]. Moreover, it has been shown that *R. officinale* root extract (ethanol extract formulated as physcion 5 g·L^−1^ aqueous solution) can be an effective alternative for known crop protection agents in the integrated and biological control of cucumber powdery mildew [13]. Furthermore, several studies have revealed its potential in aquaculture. The anthraquinone, mitigates the adverse effects of crowding stress and promotes the growth of common carp fish [14]. Additionally, it is effective against pathogenic infections in the blunt snout (*Megalobrama amblycephala*) [15].

The annual production of medicinal rhubarb roots is estimated at approximately 7000–10,000 tons [12,16]. The *R. officinale* root extract market was valued at USD 0.15 Billion in 2022 and is projected to reach USD 0.25 billion by 2030 [17]. The pharmacological activity of rhubarb root is primarily attributed to its potent laxative effects, which promote intestinal contraction and motility. These actions are mediated by the presence of different anthranoids, including anthraquinones, anthrones, and dianthrone compounds, mainly rheinosides A–D, palmidins A, B, and C, rheidins A, B, and C, as well as sennosides A–F [18]. Besides the laxative effect of *R. officinale*, different extracts and compounds originating from the roots of this plant have also been investigated in the context of other health-promoting or therapeutic actions. Research has highlighted its hypolipidemic and anti-obesity effects [19], as well as its anticancer properties [20,21,22,23], nephroprotective potential [23], antimicrobial [24], and antioxidant [25] activities. Additionally, numerous studies published in recent decades have explored the other promising bioactivities of *R. officinale* extracts, compounds, and the plant itself [26,27,28,29,30].

Despite a long history of use of *R. officinale* in traditional medicine and continued relevance in modern pharmacotherapy, the effects of its preparations on the cardiovascular system and blood physiology remain poorly characterized. The vast majority of studies on this rhubarb species have focused on extracts or compounds derived from the roots. In contrast to the better-investigated petioles of edible rhubarb species, such as *R. undulatum* L. (syn. *R. rhabarbarum*), *R. tataricum* L., and *R. rhaponticum* L., the petioles of *R. officinale*, which are waste material from root production, are often overlooked in research. Although the biological properties of non-edible rhubarb species are generally less described, their potential is starting to be noticed Although the biological properties of non-edible rhubarb species are generally less described, their potential is starting to be noticed [31]. Recent findings demonstrate that *R. officinale* petioles possess nutritional value and exhibit bioactive properties that may be relevant to anti-inflammatory effects and immune system functions [12].

The present study examines the phytochemical profile, biological activity, cytotoxicity, and gut microbiota metabolism of extracts derived from petioles and roots of *R. officinale*. We aimed to valorize the potential of petioles as a novel bioactive material and potential source of active rhubarb metabolites, as well as to evaluate the health-promoting value of both petiole and root extracts in the context of the cardiovascular system. Furthermore, utilizing *R. officinale* petioles, considered agricultural waste, aligns with the zero-waste concept, promotes efficient resource use, and protects the environment.

Considering that endothelial dysfunction and vascular inflammation represent crucial steps in the pathogenesis of cardiovascular system disorders [32,33,34], this study is focused on two aspects of anti-inflammatory action that are particularly important for cardiovascular physiology and pathophysiology of cardiovascular disorders: (1) activity of key enzymes of the arachidonic acid cascade, such as cyclooxygenase-2 (COX-2) and 5-lipoxygenase (5-LOX), and (2) modulation of the inflammatory response of leukocytes and endothelial cells. Moreover, the biocompatibility of the extracts with human leukocytes and endothelial cells was verified through cytotoxicity tests.

## 2. Materials and Methods

### 2.1. Chemicals

All reference standards used in phytochemical analysis (all analytical grade), acetonitrile LC-MS grade, formic acid MS-grade, and *tert*-butanol were purchased from Merck (Darmstadt, Germany) [35]. Methanol, *n*-hexane, and *n*-butanol, all of which were of analytical grade, were purchased from Fisher Chemical (Loughborough, UK). Ultrapure water was prepared using a Milli-Q water purification system (MerckMillipore, Darmstadt, Germany). General reagents (of analytical grade) for bioassays were purchased from Sigma-Aldrich (a part of Merck KGaA, Darmstadt, Germany), BioWest (Nuaillé, France), or local manufacturers, distributed by Hurt-Chem (Duchnice, Poland). BHI broth was purchased from bioMérieux SA (Craponne, France). Formic acid, ethanol, acetonitrile, and methanol were obtained from Avantor Gliwice, Poland.

### 2.2. Plant Material

Petioles and roots of *Rheum officinale* Baill. were donated by a local herb supplier (Kawon-Hurt, Gostyń, Poland) and were authenticated by the company representative. Petioles were collected in early summer (June 2021), whereas rhizomes were collected in late autumn (October 2021). The voucher specimens of *R. officinale*/OL/2021 and *R. officinale*/KR/2021, representing petiole and root material, respectively, have been preserved at the Department of Phytochemistry of the Institute of Soil Science and Plant Cultivation in Puławy, Poland.

#### Preparation of Extracts and High-Resolution LC-MS Qualitative and Semi-Quantitative Analyses

Rhubarb extracts were obtained following the procedures previously described in our earlier studies [35,36,37], with minor adjustments. Briefly, finely powdered plant material was extracted twice with methanol containing 0.1% formic acid in an ultrasonic bath at room temperature for 24 h under dark conditions. The resulting crude extracts were filtered, concentrated under reduced pressure, and defatted with *n*-hexane. Metabolite-enriched fractions from petioles and roots were subsequently prepared using *n*-butanol as the extraction solvent, freeze-dried, and used for further analyses. Extraction yields corresponded to 43.08% and 32.38% of dry weight for petioles and roots, respectively, while *n*-butanol fractions accounted for 4.43% and 20.25% of dry weight.

Phytochemical profiling was carried out using a Thermo Scientific Ultimate 3000 RS chromatographic system (Thermo Scientific, Bremen, Germany) equipped with a Waters Cortex T3 C18 analytical column (150 × 2.1 mm, 2.7 µm; Waters, Wexford, Ireland). The LC was coupled via a splitter to a Bruker Impact II HD quadrupole time-of-flight (QTOF) mass spectrometer (Bruker Daltonik GmbH, Bremen, Germany) and a Thermo Scientific Corona Veo RS charged aerosol detector. Data-dependent MS^2^ acquisition was performed in both positive and negative ionization modes. Metabolite signals were extracted, processed, and semi-quantified as described previously [35].

### 2.3. Cell Cultures

HUVECs (human umbilical vein endothelial cells) were isolated from freshly collected umbilical cords by collagenase type II digestion, following the protocol of Jaffe et al. [38]. Cells were used at passages 3–4. HUVECs were cultured using the MCDB-131 medium (Life Technologies, Carlsbad, CA, USA), supplemented with 10% heat-inactivated fetal bovine serum (FBS) (EURx, Rio de Janeiro, Brazil), 10 ng/mL of epidermal growth factor (EGF) (Millipore, Burlington, MA, USA), and 10 mM glutamine (Invitrogen, Carlsbad, CA, USA).

PBMCs (peripheral blood mononuclear cells) were isolated from fresh human buffy coats purchased from the Regional Center of Blood Donation and Blood Treatment in Lodz, Poland. The buffy coats originated from blood units of healthy donors and were purchased as an anonymized material. The cells were isolated using density gradient centrifugation with Lymphosep medium (BioWest, Nuaillé, France) [39]. Then, PBMCs were suspended in RPMI 1640 medium (BioWest, Nuaillé, France), and their count and viability were evaluated using an automatic cell counter (BioRad, Hercules, CA, USA) and trypan blue staining [40].

Bioethics Commission at the University of Lodz, Poland, approved the study protocol (decisions No. 15/KBBN-UŁ/III/2019 and 16 (III)/KBBN-UŁ/I/2021-22).

### 2.4. Effects of the Examined Extracts on COX2 and ALOX5 Gene Expression in HUVECs

#### 2.4.1. Total RNA Isolation and cDNA Synthesis

Total RNA was isolated using the InviTrap Spin Cell RNA Mini Kit (Stratec Molecular, Berlin, Germany), according to the manufacturer’s protocol. RNA purity was estimated spectrophotometrically using a BioTek Eon™ (Santa Clara, CA, USA) microplate reader. Samples with OD260/280 > 1.8 and OD260/230 > 1.5 were considered sufficiently pure. cDNA synthesis was performed using PrimeScript RT Master Mix (Perfect Real Time, Takara; Kusatsu, Japan), according to the manufacturer’s instructions.

#### 2.4.2. Real-Time—Quantitative PCR (RT-qPCR)

Quantitative Real-Time PCR was performed using the Eco Real-Time PCR System (Illumina; San Diego, CA, USA). The total reaction volume (10 µL) consisted of 0.2 nM of forward and reverse primers, 1 µL cDNA template, 5 µL Takara BioSYBR Green Master Mix, and 3.6 µL DNAase/RNAase-free water. The amplification conditions were as follows: an initial step at 95 °C for 30 s, followed by 40 cycles of 95 °C for 5 s and 62 °C for 30 s. Primer sequences for *COX2* and *ALOX5* were described previously [35]. *HPRT1* was used as a reference for gene expression normalization, performed according to the 2^−ΔΔCt^ method [41].

### 2.5. Evaluation of the COX-2 and 5-LOX-Inhibitory Efficiency of the Examined Extracts

COX-2 and 5-LOX activities were analyzed using the enzyme inhibitor screening kits (Cayman Chemicals, Ann Arbor, MI, USA; Abcam, Cambridge, UK, respectively). The Cyclooxygenase Colorimetric Inhibitor Screening Assay Kit (Cat. No.: 701050) measures peroxidase activity colorimetrically at λ = 590 nm, based on oxidation of *N*,*N*,*N*′,*N*′-tetramethyl-*p*-phenylenediamine (TMPD). Diclofenac (5 µg/mL) served as the reference COX-2 inhibitor, a non-steroidal anti-inflammatory drug. The 5-Lipoxygenase Inhibitor Screening Kit (Fluorometric) (Abcam, Cambridge, UK; #ab284521) quantifies the hydroperoxide generation during the lipoxygenation catalyzed by the LOX enzyme. Fluorescence was recorded at Ex/Em λ = 500/536 nm. As a positive control, zileuton (LOX inhibitor) was used (at a concentration of 0.25 µg/mL).

### 2.6. Effects of the Examined Extracts on HUVEC Viability

HUVECs were seeded onto 96-well plates at a density of 1 × 10^4^ cells/well. After 16–24 h, cells were treated with extracts from the petioles and roots of *R. officinale* at concentrations of 1–100 µg/mL for 24 h. After incubation, the cell culture medium was removed, and the wells were rinsed twice with 0.02 M phosphate-buffered saline (PBS) containing Ca^2+^ and Mg^2+^ (0.8 mM/0.4 mM, respectively). The cells were then incubated in PBS containing Ca^2+^/Mg^2+^, 5.5 mM glucose, and 0.0125 mg/mL resazurin [42]. Viability was estimated based on the ability of live cells to reduce the non-fluorescent compound resazurin to resorufin, a fluorescent product. After a 3 h incubation, resorufin fluorescence was measured (λ_ex_ = 530 nm, λ_em_ = 590 nm) using a Fluoroscan Ascent microplate reader (Thermo Fisher Scientific, Waltham, MA, USA).

### 2.7. Measurements of Cytokine Secretion from PBMCs

The examined plant extracts were added to the PBMCs suspension (1.5 × 10^6^ cells/mL, in RPMI 1640 medium, containing 10% fetal calf serum and 0.1% penicillin-streptomycin), at final concentrations of 1–50 µg/mL. The cells were seeded onto a 96-well microplate (3.75 × 10^5^ cells/well) and preincubated with the extracts for 1 h in a laboratory CO_2_ incubator (at 37 °C and 95% humidity). After the preincubation, the pro-inflammatory response of the PBMCs was induced by adding a concanavalin A (Con A; final concentration of 10 µg/mL; Sigma-Aldrich/Merck KGaA, Darmstadt, Germany) and the cells were cultured for 24 h. The next day, microplates were centrifuged to obtain supernatants (cell culture medium) for further analyses [35].

TNF-α and interleukins (IL-2 and IL-6) were detected in cell culture medium using commercial enzyme-linked immunosorbent assay (ELISA) kits from the Quantikine™ product series (R&D Systems, Minneapolis, MN, USA), i.e., TNF-α—Catalog #: DTA00D; IL-2 Catalog #: DT2050; IL-6 Catalog #: D6050B, respectively.

### 2.8. Cytotoxicity Assays in PBMCs Culture

The examined extracts were added to the PBMCs suspension (1.5 × 10^6^ mL, in RPMI 1640) to obtain their final concentrations of 1–100 µg/mL. Then, cells were seeded onto 96-well microplates (3 × 10^5^ cells/well) and cultured for 24 h (37 °C, 5% CO_2_ concentration, and 95% humidity). PBMCs treated with 0.5% Triton-X100 (Sigma-Aldrich (a part of Merck KGaA, Darmstadt, Germany) served as non-viable controls (0% cell viability).

In the resazurin reduction-based tests, after the 24 h incubation, the resazurin solution (TOX8 In Vitro Toxicology Assay Kit, Sigma-Aldrich/Merck KGaA, Darmstadt, Germany) was added to the cell culture (to the final concentration of 10%). Cell viability was determined after 4 h, using a microplate spectrophotometer (BMG Labtech SectroStarNano, Ortenberg, Germany), at λ = 600 nm (reference λ = 690 nm).

For the trypan blue dye exclusion assays, after 24 h incubation, the PBMCs suspensions (control/untreated and incubated with the examined extracts) were mixed with trypan blue and analyzed using the automatic cell counter (BioRad, Hercules, CA, USA) [43].

### 2.9. Experiments on Human Gut Microbiota

Freeze-dried *R. officinale* extracts were dissolved in deionized, sterile water to obtain a solution with a concentration of 20 mg/mL and then filtered through a 0.45 μm syringe filter. Human fecal samples were obtained from healthy volunteers (aged 25–32) with no history of gastrointestinal disease and or antibiotic use in the previous six months. Participants abstained from flavonoid- and saponin-rich foods for four days prior to collection. The study adhered to the Declaration of Helsinki and was approved by the Ethics Committee of the Medical University of Warsaw (AKBE/151/2021).

The fecal samples were processed within 30 min after defecation. The experiment was conducted in a Bactron anaerobic chamber. The inoculum (fecal slurries, FS) was prepared by suspending a fecal sample in a growth medium (brain heart infusion, BHI) (1:10, *w*/*v*; 37 °C). BHI was prepared according to the manufacturer’s instructions. BHI was boiled and then immediately cooled to achieve anaerobic conditions before the experiment. The tested samples were prepared appropriately: 1 mL of extract, 1 mL of FS suspension in BHI, and 8 mL of BHI. As a control, incubations of extract without FS and FS without extract in BHI were performed. The batch cultures were incubated in a sealed container under anaerobic conditions at 37 °C. Incubations were terminated after 0, 2, 5, 8, and 24 h. Samples were collected for analysis by taking 0.5 mL of the mixture and then mixing it with 0.5 mL of methanol with 0.1% formic acid. Samples were then centrifuged (5 min, 9000 RPM) and the supernatants were filtered through a 0.45 μm syringe filter (PVDF) and analyzed using UHPLC-DAD-MS^n^.

### 2.10. Chromatographic Analysis of Extracts Metabolized by Human Gut Microbiota

UHPLC-DAD-MS^n^ analysis was performed using the UHPLC-3000 RS system (Dionex, Leipzig, Germany), equipped with a DAD detector and splitless connection with an AmaZon SL ion trap mass spectrometer with an ESI interface (Bruker Daltonik GmbH, Bremen, Germany). The UV spectra were recorded in the wavelength range of 200–450 nm. The parameters of the MS unit were as follows: nebulizer pressure, 40 psi; drying gas flow rate, 9.0 L/min; nitrogen gas temperature, 300 °C; and capillary voltage, 4.5 kV. The mass spectra were registered by scanning from *m*/*z* 70 to 2200. A Kinetex XB-C18 (150 mm × 2.1 mm × 1.7 μm) chromatography column was used (Phenomenex, Torrance, CA, USA). The mobile phase (A) was H_2_O/HCOOH (100:0.1, *v*/*v*), and the mobile phase (B) was MeCN/HCOOH (100:0.1, *v*/*v*). The gradient program and flow rate were as follows: 0–60 min, 1–26% B; 60–80 min, 26–95% B, with a constant flow rate of 0.3 mL/min. The column oven temperature was set to 25 °C. Before each chromatographic analysis, samples were filtered.

### 2.11. Statistical Analysis

The statistical analysis was performed using the STATISTICA 13.0 PL software (StatSoft Inc., Tulsa, OK, USA). Uncertain data were eliminated by the Grubbs’ tests (GraphPad Prism 5.01, San Diego, CA, USA). The normality of the data distribution was assessed using the Shapiro–Wilk test. Based on the results, an appropriate statistical test (parametric or non-parametric) was selected. For a normal distribution, the *t*-test for dependent samples was applied. In the absence of a normal distribution, the Wilcoxon signed-rank test was used. The *p* < 0.05 values were considered statistically significant. The letter “*n*” in figure legends and table captions refers to the number of independent donors/experiments. Typically, the samples were measured in duplicate or triplicate to ensure the technical reliability of the analysis.

## 3. Results

### 3.1. Phytochemical Profile of the Petiole and Root Extracts of R. officinale

**Figure 1:**
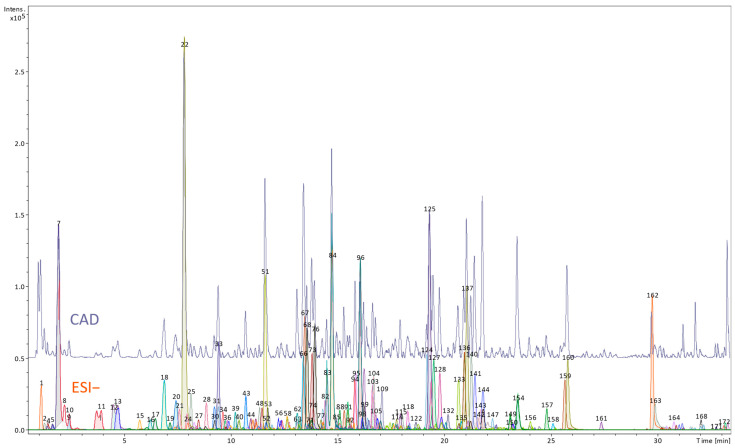
UHPLC profile of the butanol extract obtained from *Rheum officinale* roots (upper panel—CAD detector signal, lower panel—MS chromatogram using negative ESI mode; the numbers correspond to the numbers of compounds tentatively identified in Table S1).

**Figure 2:**
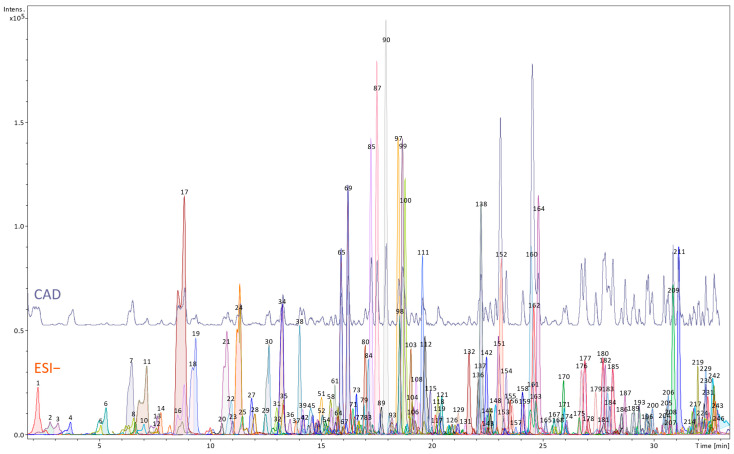
UHPLC profile of the butanol extract obtained from the petioles of *Rheum officinale* (upper panel—CAD detector signal; lower panel—MS chromatogram using negative ESI mode; the numbers correspond to the numbers of compounds tentatively identified in Table S1).

Table S1 (Supplementary Materials) and Figure 1 and Figure 2 show phytochemical profiles of the root and petiole extract of *R. officinale*, including the identification results and semi-quantitative data on the contents of selected metabolites.

As shown in Figure 1, chromatographic analysis of the root extract of *R. officinale* resulted in the detection of 174 metabolites at varying levels of identification (Table S1). Anthraquinones were the most prevalent compound class, accounting for a 23% share of detected metabolites. However, to our surprise, they were less diverse (40 vs. 57 compounds) and less abundant than in the petiole extract (total content estimated at 92.2 ± 7.6 µg/mg dry weight (dw)). The root extract contained sennosides (dianthrones of rhein or mixed dianthrones of rhein and aloe-emodin), several derivatives of chrysophanol, physcion, and rhein, as well as fewer than petioles metabolites of emodin (only a few peaks of emodin dianthrones were present). The most abundant class responsible for this difference was phenolic acid derivatives (24% of detected metabolites, estimated to be 136.1 ± 2.3 µg/mg dw). Most of these were hexosyl gallate esters, typically accompanied by other phenolic acids, stilbenes, and hydroxybenzylphenones. Compared to petioles, more catechins were observed (8% of all detected compounds), and their total contents were substantially larger (102.6 ± 6.8 µg/mg dw). Chromone derivatives and flavonoids were also observed (7 and 6% of detected compounds count, respectively), although their contents were relatively low (estimated at 13.2 ± 0.5 and 10.2 ± 0.4 µg/mg dw), significantly lower than in petioles. Stilbenes, all derived from trans-resveratrol, were represented by fewer compounds than detected in petioles but were much more abundant (estimated at 34.1 ± 0.9 µg/mg dw). Torachrysone (naphthol) derivatives were almost equally abundant (estimated at 35.5 ± 1.2 µg/mg dw). Among amino acid group, both tyrosine and tryptophan were detected in roots. However, their combined content was estimated at only 1.5 ± 0.1 µg/mg dw, nearly ten times lower than in petioles. The root extract also contained organic acids and carbohydrates, estimated at 5.9 ± 0.1 and 21.0 ± 1.2 µg/mg dw, respectively.

**Figure 3:**
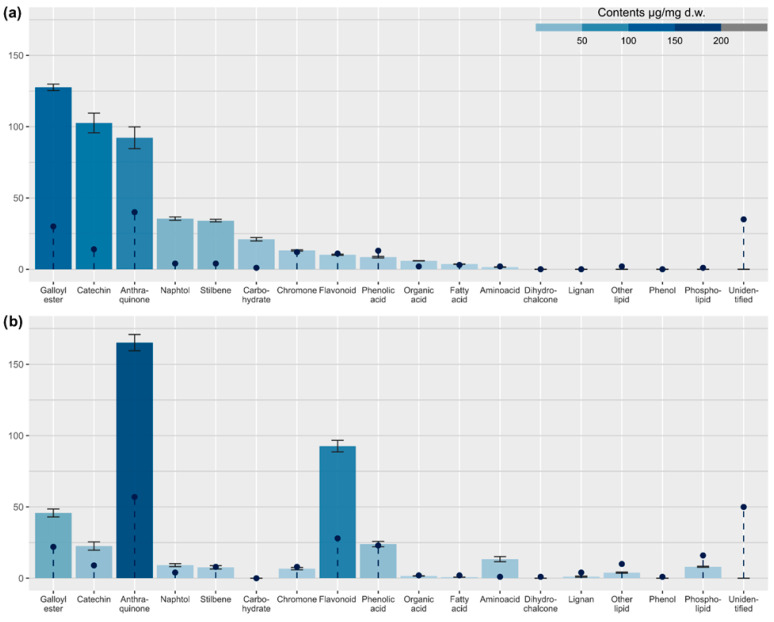
Comparison of metabolite distribution between *R. officinale* roots (**a**) and petioles (**b**) extracts. Bars represent estimated contents (with standard deviations (*n* = 3) as error bars). Dashed lines with dots represent the number of compounds identified in each class.

A higher number of compounds, 246, was observed in the petiole extract (Figure 2, Table S1). The primary class of fully or partially identified metabolites was the group of anthraquinone derivatives (23% of detected compounds, estimated at 165.1 ± 5.7 µg/mg dw in total). These metabolites were mainly derived from emodin. Only a few metabolites of physcion and none of chrysophanol were observed. However, a wide variety of isomeric emodin dianthrones was revealed, indicating significant complexity of anthraquinone metabolism in petioles. Anthranoids were accompanied by other metabolites, recognized as phenolic acid derivatives (18%, half of that were hexosyl gallates containing in their structures another phenolic acid, phenol or stilbene moiety; total phenolic acid contents estimated at 69.8 ± 3.4 µg/mg dw), flavonoids (11%, 92.7 ± 4.1 µg/mg dw), polar lipids, including phospholipids (10%, estimated total contents 12.7 ± 0.6 µg/mg dw), as well as stilbenes, chromone and catechin derivatives (approx. 3% of detected compounds in each class, estimated at 7.7, 6.7, and 22.6 µg/mg dw, respectively). All detected stilbenes were derivatives of trans-resveratrol. Naphthol derivatives and lignans (both classes, accounting for 1.6% of the total detected compounds, estimated at 9.2 and 1.1 µg/mg dw, respectively) were also identified. The free amino acids were only represented by tryptophan, which was relatively abundant (13.4 ± 1.8 µg/mg dw) in the extract (Figure 3).

### 3.2. Effects of the Examined Extracts on COX2 and ALOX5 Gene and Protein Expression in HUVECs

**Figure 4:**
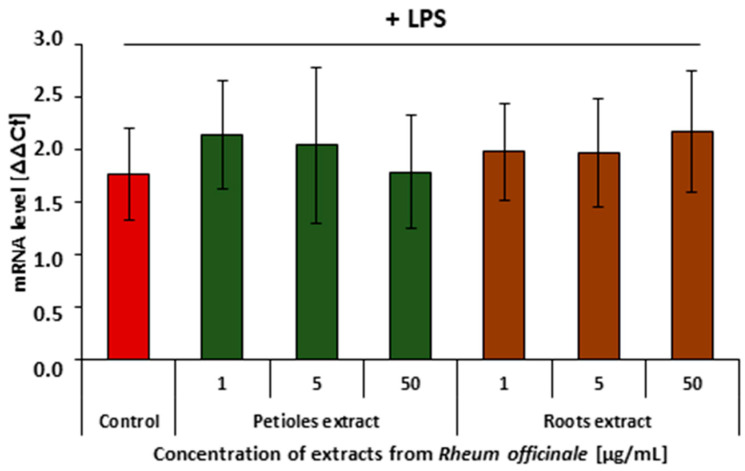
Effects of the *R. officinale* extracts on the *COX2* gene expression. Data from experiments involving HUVECs treated with the examined extracts, followed by stimulation with LPS. Data are presented as mean values ± SD; *n* = 7.

**Figure 5:**
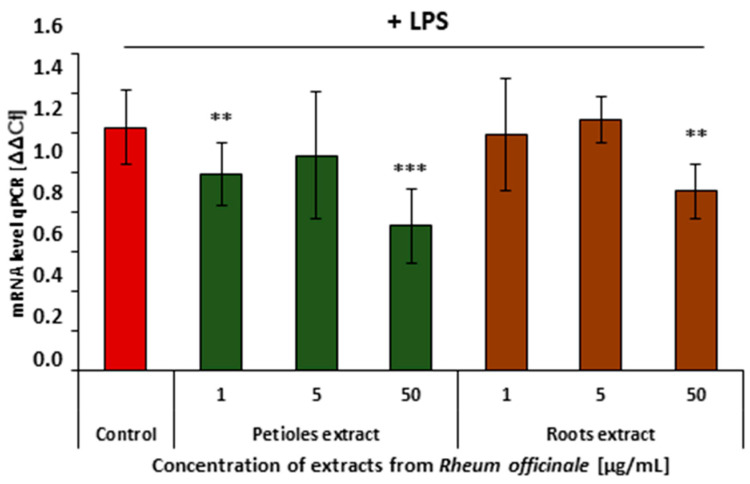
Effects of the *R. officinale* extracts on the *ALOX5* gene expression. Data from experiments involving HUVECs treated with the examined extracts, followed by stimulation with LPS. Data are presented as mean values ± SD; (** *p* < 0.01; *** *p* < 0.001); *n* = 7.

The COX-2 and 5-LOX enzyme-mediated arachidonic acid metabolism is considered one of the key steps of cell inflammatory response. RT-qPCR analysis revealed that neither the *R. officinale* petiole nor root extract affected *COX2* gene expression (Figure 4). However, both examined extracts partly suppressed *ALOX5* gene expression (Figure 5).

### 3.3. Evaluation of the COX-2 and 5-LOX-Inhibitory Ability of the Examined Extracts

**Figure 6:**
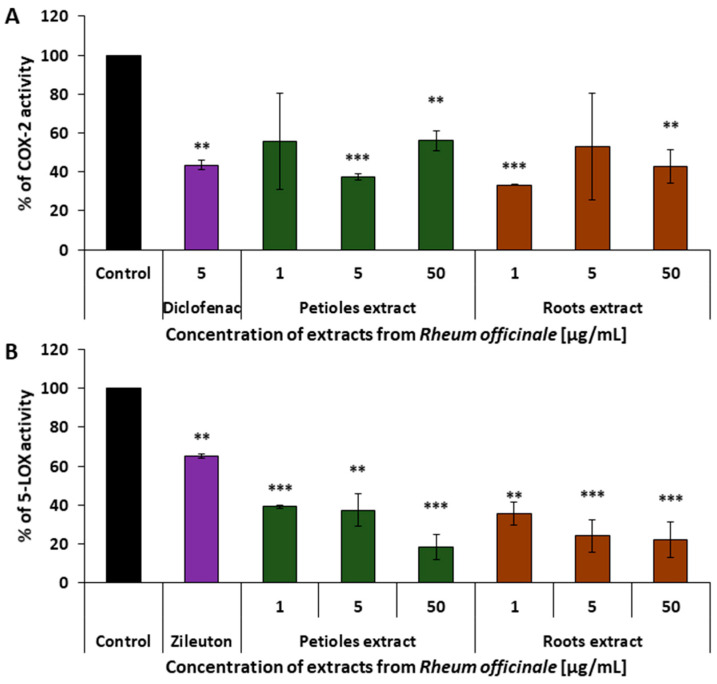
Inhibitory effect of petiole and root extracts of *R. officinale* on the activity of pro-inflammatory enzymes: (**A**) inhibition of the enzymatic activity of COX-2. Diclofenac (5 µg/mL)—a reference COX inhibitor; (**B**) inhibition of the enzymatic activity of 5-LOX. Zileuton (0.25 µg/mL)—a reference 5-LOX inhibitor. The activity of native enzymes (untreated with any of the examined extracts) was assumed as 100%; *n* = 7 (** *p* < 0.01; *** *p* < 0.001).

At the enzymatic level, the anti-inflammatory effects of the examined extracts were evaluated by measuring their inhibitory activity on COX-2 and 5-LOX, using colorimetric and fluorometric assays, respectively. Both extracts demonstrated comparable inhibitory effects on COX-2 activity, with maximum inhibition exceeding 60%. No clear dose dependence was observed within the examined concentration range (1–50 µg/mL). The inhibitory potency of both extracts was equal to or greater than that of diclofenac (5 µg/mL) (Figure 6A).

### 3.4. Effects of the Examined Extracts on HUVECs Viability

**Figure 7:**
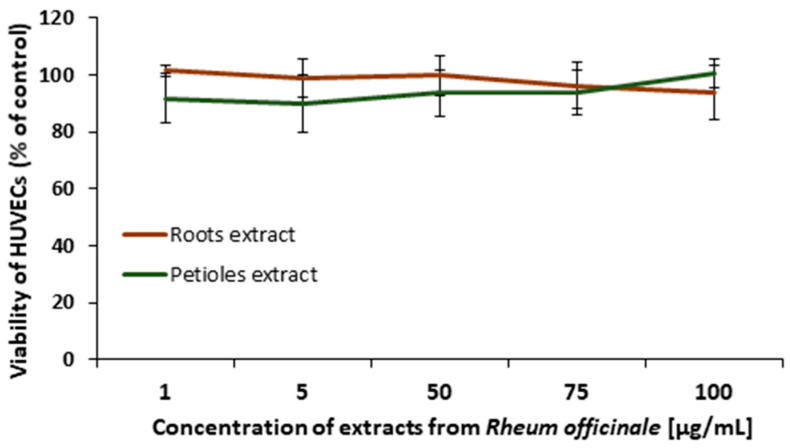
Effects of the examined extracts on the HUVECs viability. The figure presents viability curves of HUVECs after 24 h of incubation with petioles or root extracts from *R. officinale*. Metabolic activity of the cells was established based on resazurin reduction (0.0125 ng/mL); λ_ex/em_ = 530/590. Data are presented as mean values ± SD; *p* > 0.05; *n* = 3.

The viability of HUVECs treated with the examined extracts (1–100 μg/mL) for 24 h was evaluated based on the resazurin metabolic test. No significant decrease in cells viability was observed (*p* < 0.05; Figure 7), indicating the cellular safety of both *R. officinale* extracts.

### 3.5. Effects of the Examined Extracts on the Inflammatory Response of the PBMCs

**Figure 8:**
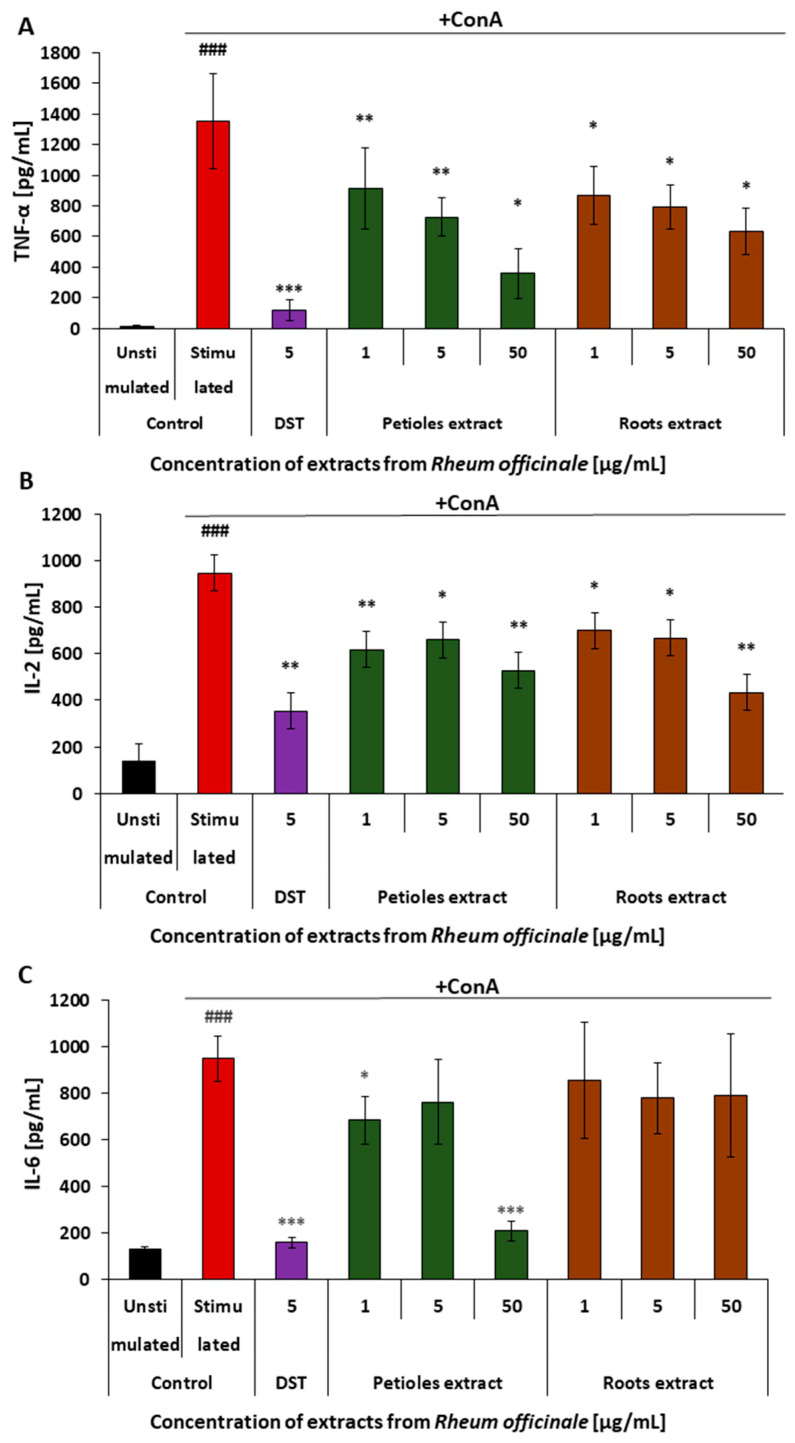
Effects of the examined petiole and root extracts from *R. officinale* on the inflammatory response of human PBMCs. The anti-inflammatory effects of the examined extracts were determined based on the TNFα (**A**), IL-2 (**B**), and IL-6 (**C**) release from the concanavalin A-stimulated PBMCs. The cytokine level in the cell culture medium was quantified by the ELISAs. DST (dexamethasone, a steroid anti-inflammatory drug) was used as a reference compound. The figure represents mean values (±SD); ^###^ *p* < 0.001 for unstimulated PBMCs vs. PBMCs treated with Con A in the absence of the examined extracts; the cytokine (pg/mL) detected in samples derived from the Con A-stimulated cells and treated with the examined extracts vs. cells treated in the absence of the examined extracts: * *p* < 0.05, ** *p* < 0.01, *** *p* < 0.001; *n* = 4.

The anti-inflammatory action of the examined *R. officinale* extracts in PBMCs was determined based on the level of cytokines (i.e., TNF-α, IL-2, and IL-6) secreted from these cells in response to a pro-inflammatory stimulation by Concanavalin A (Figure 8). The obtained results indicated the ability of the extracts to reduce the inflammatory response of PBMCs. Although the petiole and root extract displayed comparable effectiveness in the case of TNF-α and IL-2 release, their ability to suppress the IL-6 release considerably differed. Both extracts reduced cytokine secretion, indicating the ability to attenuate PBMC inflammatory responses. While the extracts showed comparable efficacy in suppressing TNF-α and IL-2 release, significant differences were observed in IL-6 modulation. The petiole extract markedly reduced IL-6 secretion, achieving over 70% reduction, whereas the root extract had no statistically significant effect on this cytokine.

### 3.6. Cytotoxicity Evaluation in PBMCs

**Figure 9:**
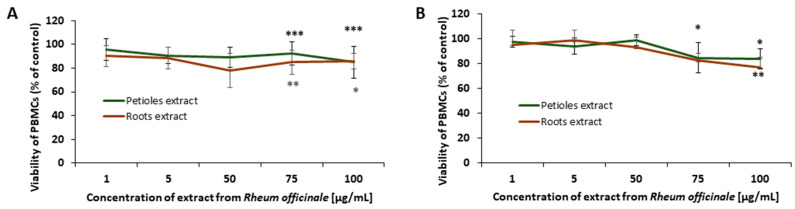
Evaluation of the *R. officinale* extracts cytotoxicity in PBMCs. The viability curves of PBMCs after 24 h of incubation with petioles and root extracts from *R. officinale* were prepared based on the results of the resazurin reduction and the trypan blue exclusion tests. The viability of cells untreated with the extracts was assumed to be 100%. Data are presented as mean values ± SD; (* *p* < 0.05; ** *p* < 0.01; *** *p* < 0.001); *n* = 11.

The risk of cytotoxic action of the examined extracts towards PBMCs was assessed using two different tests (Figure 9). The trypan blue dye exclusion test was applied to evaluate the effects of *R. officinale* extracts on cell membrane integrity Figure 9B. In addition, the effects of the examined extracts on PBMCs viability were analyzed using the resazurin metabolic test Figure 9A. No effects of the *R. officinale* extracts on PBMCs membrane integrity and their viability were found in the concentration range of 1–50 μg/mL. However, at higher concentrations (i.e., 75 and 100 μg/mL), a slight decrease in cell viability (by about 20–25%) was observed.

### 3.7. Metabolism of R. officinale Extracts by Human Gut Microbiota

Extracts from the petioles and roots of *R. officinale* were incubated (2, 5, 8, and 24 h) in a growth medium BHI, with human gut microbiota (FS). The concentration of extracts in the tested samples was 2 mg/mL. Sample controls include BHI + FS (*v*/*m*) and BHI + extracts (*v*/*v*). The analysis focused on the main compounds identified in *R. officinale* extracts: derivatives of (aloe)emodin, rhein, chrysophanol, and physcion, including sennosides and other dianthrones (Supplementary Material Table S1). A tentative structural identification of their metabolites M1-M9 formed during FS-stimulated digestion was performed using a UHPLC-DAD-MS^n^ method by comparing their spectral profiles with literature data and those of reference standards [44,45,46].

The first process observed in the batch culture with an extract from petioles was the formation of (aloe)emodin (M1) after 2 h of incubation. It eluted at 73.8 min (Figure S1), with the deprotonated molecule [M–H]^−^ at *m*/*z* 268.87 (Table 1). The presence of M1 suggested the hydrolysis of native (aloe)emodin glycosides and their malonyl esters, which may be represented by those detected as peaks no. 112, 141, and 162 in the crude extract (Table S1, chart: Petioles). The subsequent hydroxylation (+16 Da) and acetylation (+42 Da) of the liberated emodin led to the creation of hydroxy-emodin (M2) and acetyl-hydroxy-emodin (M3), respectively. M2 and M3 eluted at 62.2 and 71.3 min (Figures S2 and S3), with the deprotonated molecules [M–H]^−^ at *m*/*z* 285.05 and 327.4. In the same incubation time, hexose units (162 Da) and malonyl moieties (86 Da) were cleaved from (aloe)emodin-physcion-dianthrone-malonyl dihexosides (Table S1, peaks 174, 186, 190, 193, 197, 199). It resulted in the presence of (aloe)emodin-physcion-dianthrone-hexoside (M4), detected at 72.0 min (Figure S4), with the [M–H]^−^ ion at *m*/*z* 685.30. The most extended incubation with FS (at least 5 h) was required for the oxidative cleavage of these dianthrones or physcion anthrones/glycosides (e.g., peaks 171, 183, and 184, Table S1), and further metabolization of the liberated physcion through demethylation (−14 Da) and subsequent dehydroxylation (−16 Da). The obtained chrysophanol isomer (M5) was eluted at 11.5 min (Figure S5) and detected with the ion [M–H]^−^ at *m*/*z* 253.26. Simultaneously, further amounts of M1 were produced through the cleavage of (aloe)emodin dianthrones (e.g., peaks no. 154, 160, 176, 177, 182, and 185, Table S1), which was evidenced by markedly increasing content of this metabolite after 5 h and 8 h of incubation [44,45,46].

In the mixture extract from the roots of *R. officinale* and FS, four metabolites M6-M9 of emodin, rhein, and chrysophanol derivatives, including sennoside A and sennosides C/D, were detected (Table 1). Metabolite M6 (Figure S6) was recorded after 2 h of incubation and confirmed as rhein based on its retention time (71.0 min), [M–H]^−^ ion at *m*/*z* 282.96, and fragment ion at *m*/*z* 238.74. It was formed either by hydrolysis of rhein glycosides (e.g., peaks 73 and 99, Table S1, chart: Roots) or as the oxidized product of rheinanthrone, liberated from rhein dianthrone-glycosides (e.g., peaks 85, 90, and 105, Table S1) after their hydrolysis and cleavage of the C10–C10′ bonds. Therefore, the level of M6 increased constantly after 5 h, 8 h, and 24 h of incubation. Metabolite M7 was detected after 5 h of incubation. It eluted at 66.0 min (Figure S7), with the deprotonated molecule at *m*/*z* 313.15, and the fragment ion at *m*/*z* 268.75. Following the literature, M7 was identified as acetyl-1,3,8-trihydroxy-6-methyl-9-oxanthranol or acetyl-1,3,8-trihydroxy-6-methyl-10-oxanthranol, a typical metabolite of emodin [45]. Considering the profile of the crude root extract (Table S1), M7 might be formed from some anthranoids identified as (aloe)emodin glycosides (e.g., peaks 66 and 136, Table S1) by hydrolytic cleavage of their sugar units and subsequent hydrogenation and acetylation of free (aloe)emodin. M7 might also be produced by hydrolysis of chrysophanol glycosides (e.g., peaks 128, 141, 144, and 149), hydroxylation of the liberated compound to emodin, and its subsequent metabolization following the reactions described above. Two further detected metabolites M8 and M9 originated from sennosides. M8 was eluted at a retention time of 65.8 min (Figure S8), with the deprotonated molecule at *m*/*z* 699.22 [M–H]^−^ and fragment ions at *m*/z 537.07 and 223.25, typical for sennidin A-8-*O*-glucoside. It was created after 2 h of incubation from sennoside A or its malonyl esters (peaks 80, 90, and 105, Table S1) by hydrolysis and loss of one sugar moiety (−162 Da) and malonyl unit (−86 Da). M9 eluted at 64.8 min (Figure S9) as a result of analogous transformation of sennosides C/D (peaks 70, 79, 85, and 113, Table S1) into sennidin C/D-8-*O*-monoglucose/sennidin C/D-8′-*O*-monoglucoside [44,45,46].

## 4. Discussion

Numerous pharmacological effects of *R. officinale* root, which contribute to its ability to treat a broad spectrum of ailments and have other actions and applications in agriculture and various industry branches, as detailed in the Introduction, have attracted attention and contributed to its popularization in many Western countries. Our comprehensive approach to the *R. officinale* was based on chromatographic fingerprinting, human gut microbiota-assisted digestion, and different biochemical/molecular biology in vitro examinations. We conducted a comparative analysis of the phytochemical and biological activity profiles of extracts from the roots and petioles of *R. officinale*, focusing on its potential to modulate inflammatory processes using experimental systems related to cardiovascular physiology.

The growing demand for rhubarb, in turn, leads to its excessive extraction/overharvesting from natural sources, which may result in environmental damage and contribute to its inclusion on the list of endangered species [47,48,49]. Therefore, the agricultural cultivation of rhubarb significantly increased, and some good agricultural practices (GAP) have been established for the genuine species in China [11] and outside its natural place of origin, including many European countries [1]. However, the large-scale cultivation of medicinal rhubarb generates vast amounts of waste material, specifically the above-ground parts. Data confirming the nutritional value of *R. officinale* petioles [12] have initiated a new direction in studies on the plant’s potential in an industrial context and prospects for new functional applications. Moreover, such research aligns with the zero-waste concept, which is also crucial in agricultural practice, promoting the efficient use of resources and protecting the environment. Agri-food waste contains valuable organic compounds that can be reused in value-added products, promoting sustainable waste management practices in line with the UN Sustainable Development Goals (SDGs) [50,51,52,53]. Moreover, converting rhubarb’s petioles offers a potential way to increase farmers’ incomes while reducing agricultural waste.

Among the various classes of compounds essential for the therapeutic effectiveness of *Rhei* radix, the main active ingredients and quality markers are derivatives of anthraquinones, including rhein, emodin, aloe-emodin, chrysophanol, and physcion. Although the content of anthraquinones may vary significantly depending on the species, cultivation conditions, and processing methods, their overall content confirms the quality of plant material and extracts [54,55,56,57]. The Chinese Pharmacopeia specifies that the total anthraquinone content in rhubarb should not be less than 1.5%. In comparison, the Japanese Pharmacopeia requires the sennoside A content to be higher than 0.25% [5,58,59]. The European Pharmacopeia requires a content of not less than 2.2% of hydroxyanthracene derivatives, expressed as rhein [60]. As demonstrated in Figure 3 and Table S1, the estimated total anthranoid content in the petiole and root extracts is 16.5% dry weight (dw) and 9.2% dw, respectively, meeting the pharmacopeial quality criteria unequivocally. Notably, the anthranoid content in the petiole extract is nearly double that found in the root extract. It exceeds the official standard by a factor of four, despite significant qualitative differences between the two extracts.

Determining the quality of the raw material is a crucial element in the therapy used and its effectiveness. Chromatographic fingerprinting, which combines liquid chromatography with high-resolution mass spectrometry, is commonly recommended as an effective strategy for identifying and controlling the quality of various plant materials and herbal preparations. Such an analysis has also been employed to characterize the profile of *R. officinale* metabolites in the present study, as well as in some previous studies [8,56,57,61,62].

Diverse external environmental factors, such as rainfall levels, light intensity, ultraviolet radiation, temperature, altitude, soil humidity, fertility, or salinity, influence the biosynthetic pathways of specialized metabolites in medicinal plants, leading to fluctuations in their content and accumulation [63,64,65]. For this reason, while plant material originating from different places usually exhibits similar qualitative profiles of metabolites, significant quantitative differences have often been reported [56,57,66,67]. The quantitative analysis of multi-component extracts, such as rhubarb extracts, poses challenges due to the absence of suitable reference standards. As a result, quantitative determinations are often limited to selected compounds for which either commercial or self-isolated/synthesized standards [54,55,57,59,68,69]. Another possibility is to determine only the content of aglycones after acid hydrolysis, as recommended by the official regulations regarding anthraquinone determination [55,56,60,70]. Quantitative analysis of multiple components by a single marker (QAMS) can also be performed using only one reference substance and relative correction factors for assessing other [56,71,72,73,74,75]. As shown in Table S1, in this work, we applied semi-quantification based on the responses of 32 metabolites from a charged aerosol detector [35,76]. Due to the specific features of this type of detection [77], direct comparisons between samples analyzed under the same conditions are undoubtedly valid and accurate; however, the estimated contents of the detected metabolites should be regarded as approximate.

Literature data indicate that the quantitative profile of official medicinal rhubarb roots depends on the plant species and habitats. Significant variations in total anthraquinone glycoside content, reaching even up to 35-fold between the examined samples, were observed [59]. The highest level (mean ± SD) was recorded for samples of *R. tanguticum* (46.21 ± 19.25 mg/g), followed by *R. palmatum* (35.54 ± 22.22 mg/g). In contrast, samples of *R. officinale* roots were characterized by the lowest concentration of anthraquinone glycosides (26.30 ± 22.98 mg/g). Moreover, the content of all examined anthraquinone glycoside varied greatly, i.e., the aloeemodin-8-*O*-glucoside content ranged within 1.74–18.33 mg/g, for rhein-8-*O*-glucoside 1.87–35.55 mg/g, for emodin-1-*O*-glucoside 0.45–18.61 mg/g, for chrysophanol-1-*O*-glucoside 1.72–17.03 mg/g, for chrysophanol-8-*O*-glucoside 1.87–46.99 mg/g, and finally 0.42–18.33 mg/g for emodin-8-*O*-glucoside. Furthermore, results clearly demonstrate that altitude affects the biosynthesis and accumulation of anthraquinone glycosides, primarily the contents of aloeemodin-8-*O*-glucoside, emodin-1-*O*-glucoside, emodin-8-*O*-glucoside, and chrysophanol-1-*O*-glucoside. Rhubarb samples collected at low altitudes accumulated significantly lower amounts of anthraquinone glycosides, which may affect the clinical effects of the preparations obtained from such medicinal material. In rhubarb samples collected below 2500 m, the total anthraquinone glycoside content was only 24.25 ± 20.19 mg/g, which was less than half of that observed in samples collected above 2500 m (45.12 ± 18.55 mg/g). These results suggest that samples collected below 2500 m may be inferior as a source of rhubarb for pharmaceutical preparations. Therefore, they should be administered in double the usual dose to ensure their therapeutic effectiveness.

Ye and colleagues highlighted the taxonomic significance of specific metabolites in classifying rhubarb [61]. The same sennosides, anthraquinone glycosides, and glucose gallates were detected in samples of *R. palmatum* and *R. tanguticum*, while a completely different profile was observed for *R. officinale*. Based on information obtained from MS analyses, researchers concluded that the differences in chemical composition are related to the presence of isomers in different species: sennoside A was observed only in *R. officinale.* However, its isomers were detected in large amounts in the other two official species. Similarly, it was found that the predominant anthraquinone glycosides in *R. officinale* were rhein 8-*O*-glucoside and emodin 1-*O*-glucoside, while in *R. palmatum* and *R. tanguticum* were rhein 1-*O*-glucoside and emodin 8-*O*-glucoside.

Based on qualitative UHPLC-QTOF-MS analyses and semi-quantitative UHPLC-CAD metabolite determination, we extensively characterized the phytochemical composition of the root and petiole extracts of *R. officinale*. Similarly to previous studies on *R. officinale* roots [8,56,57,61,62], our analysis confirmed the presence of compounds from multiple classes, including anthraquinones, anthrones, flavonoids, phenolic acids, stilbenes, and other bioactive metabolites. Compared to previously studied rhubarb species [35], we did not observe the known metabolites of either rhapontigenin or piceatannol in the two investigated extracts of *R. officinale*. Following the level of anthranoid content mentioned earlier, the second quality criterion listed in pharmacopeias for rhubarb roots refers to the exclusion of the phytoestrogenic rhaponticin (syn. rhapontin), a glucoside of rhapontigenin. It is generally accepted that rhubarbs from the *Palmata* section do not contain rhaponticin, which is considered a potential marker for unofficial rhubarbs from the section *Rhapontica* (*Rheum rhaponticum* L. and *Rheum rhabarbarum* L.; syn. *R. undulatum* L.). This distinction is strongly emphasized by both European and Chinese Pharmacopeias—in the assay for medicinal rhubarb purity, rhaponticin should not be detectable by thin-layer chromatography (TLC) [58,61,70,78,79,80].

On the other hand, the metabolites of trans-resveratrol were detected, indicating that, while the stilbenoid biosynthesis pathway may not be fully operational in *R. officinale*, the plant can still synthesize some stilbenoids. Nevertheless, based on our semi-quantitative results, it seems that the activity of glucosyltransferase forming 4′-*O*-stilbenoid glucosides is functional in *R. officinale* (resveratroloside accumulated to a similar level as in garden rhubarbs). In contrast, the activity of 3-*O*-glucosyltransferase is almost entirely missing. Thus, only trace levels of piceid/polydatin are observable. Furthermore, oligostilbenes-products of the oxidative condensation of the stilbene monomers are also missing from the extracts of *R. officinale*. The lack of oligostilbenes is puzzling, given that resveratrol was produced and, as mentioned earlier, 4′-*O*-glucoside was present in the roots. The lack of both piceid and oligostilbenes may be purely coincidental. However, it may also indicate some functional connections, for example, piceid may serve as a precursor for oligostilbene biosynthesis.

Few studies have addressed *R. officinale* petioles, despite their occasional consumption by local populations [22,81]. Unfortunately, the existing reports are of limited quality in terms of phytochemical composition analysis. For example, in the work of Dai and colleagues [22] on petioles of *R. officinale* and *R. tanguticum*, the only metrics used for identification were accurate mass measurements and calculated elemental formulas. Spectral comparison with standards or interpretation of fragmentation patterns for the investigated analytes was not performed. Moreover, classifying and grouping compounds based on such relatively imprecise identification was somewhat debatable. For instance, several amines (spermidine, tryptamine, tyramine, or putrescine derivatives) have been classified as alkaloids. Furthermore, in contrast to this study, the analyzed extracts from *R. officinale* petioles contained rhapontigenin (the aglycone of rhaponticin), isorhapontigenin, and deoxyrhapontin (Table S1 in the Supplementary Materials).

Based on our results, the petiole extract of *R. officinale* generally contained a greater diversity of phenolic compounds, primarily flavonoids and phenolic acid derivatives. In the case of anthranoids, in the roots, compounds such as emodin and aloe-emodin are transformed into other metabolites, including chrysophanol, physcion, and rhein, which then lead to the formation of rhein dianthrones (sennosides). In contrast, the predominant metabolic process in the petioles involves the formation of various isomeric aloe-emodin dianthrones, with very few other anthraquinone metabolites detected. Therefore, *R. officinale* petioles may serve as an interesting source of active compounds, particularly given the lengthy and challenging process of obtaining high-quality root material. However, further research is necessary to investigate the activity of emodin-dianthrone glycosides and determine the potential applications of petiole extracts in the industry.

Interestingly, the levels of flavonoids in petioles are nine times higher than those in the roots. Flavonoids are known to be potent antioxidants and anti-inflammatory agents with well-documented health benefits for chronic human disorders such as cardiovascular diseases. The most abundant flavonoids in petioles include primarily flavonols (specifically quercetin glycosides), along with flavones and flavanones (such as derivatives of apigenin, naringenin, and pinocembrin).

Furthermore, the petiole extract has three times lower levels of galloyl esters (mostly hydrolyzable tannins). High levels of these compounds are associated with an increased risk of hepatotoxicity when administered repeatedly. Therefore, based on the observed phytochemical profiles, the extracts from the petioles of *R. officinale* warrant further investigation, particularly regarding their potential benefits for cardiovascular health. Despite many ethnomedicinal recommendations and the presence of different rhubarb species in contemporary medicine, reports on their effects on blood components or cardiovascular physiology are limited. The present study is a part of our research aimed at assessing the cardioprotective potential of extracts from various rhubarb species. Previous experiments provided promising data on the antioxidant, anti-inflammatory [35,37], and anticoagulant actions [36] of two other rhubarb species, namely *R. rhaponticum* and *R. rhabarbarum*, which belong to the *Rhapontica* section. This work focuses on the phytochemical profile and anti-inflammatory activity of extracts derived from the petioles and roots of *R. officinale*, a member of the *Palmata* section. Due to the crucial role of endothelial cells and leukocytes in the inflammatory response in the cardiovascular system, the extracts’ anti-inflammatory properties were examined using two experimental models, i.e., HUVECs and human PBMCs. A particular emphasis was placed on the possibility of inhibiting COX-2 and 5-LOX, two key enzymes in the arachidonic acid cascade, responsible for synthesizing pro-inflammatory eicosanoids (prostaglandins and leukotrienes, respectively). Inhibition of these oxygenases is a major therapeutic approach targeting inflammation. However, many of the most commonly administered COX-2 inhibitors, belonging to the group of non-steroidal anti-inflammatory drugs (NSAIDs), display limited selectivity and have a risk of serious side effects [82,83,84]. Treatment with zileuton, a 5-LOX inhibitor, was reported to evoke adverse effects as well [85]. Due to the above-mentioned risk of using NSAIDs, the search for safer substances is still ongoing, and substances of natural origin are gaining much attention.

Although inflammation is a natural element of animal (incl. human) physiology and one of the adaptive response mechanisms, an uncontrolled inflammatory process (especially at a systemic level) may result in many disorders. Inflammatory processes, along with oxidative stress, endothelial dysfunction, and disorders in the hemostatic balance of the blood, are among the most important factors contributing to the development and progression of cardiovascular diseases (CVDs) [86,87,88,89,90]. Maintaining the physiological balance within the vascular system results from complex interactions between blood components and the vessel wall, with a critical role in the vasoactive functions of the endothelium. Due to the regulatory activity of the endothelium, its dysfunction triggers subsequent unfavorable changes in the physiology of the blood and the cardiovascular system [91,92]. Anti-inflammatory activity of plant extracts and isolated compounds may involve diverse mechanisms and different molecular/cellular levels, including the inhibition of the nuclear factor *kappa*-light-chain-enhancer of activated B cells (NF-κB) activation, suppression of the phosphatidylinositol 3′-kinase (PI3K)/Akt signaling pathway activation, upregulation of the sirtuin 1 (SIRT1) expression, inhibition of adhesion molecules expression and their functions, reduction in cytokine release or direct inhibition of pro-inflammatory enzymes [93,94]. Among the above mechanisms, inhibition of pro-inflammatory oxygenases in the arachidonic acid cascade is one of the essential medical approaches. Moreover, the pro-inflammatory pathways mediated by COX and LOX enzymes still belong to the most widely studied targets for anti-inflammatory therapy [95].

This study provides the first data on the effects of the *R. officinale* extracts on the inflammatory reactivity of human endothelial cells and leukocytes at different molecular levels. Our results indicated that both of the examined extracts can partly inhibit the metabolism of arachidonic acid in endothelial cells. Their inhibitory, and consequently anti-inflammatory, action was more pronounced in lowering the activities of COX-2 and 5-LOX enzymes than at the level of *COX2* and *ALOX5* gene expression. Moreover, analyses of gene expression revealed significant divergences in the up-regulatory action of the examined extracts. Although neither the examined petiole nor the root extract affected the *COX2* gene expression, both of them partly suppressed the *ALOX5* gene expression in HUVECs (Table 2). In contrast, our previous studies on *R. rhaponticum* and *R. rhabarbarum* extracts showed that petiole extracts significantly reduced the *COX2* expression—by more than 70%. While the petiole extracts from *R. rhaponticum* and *R. rhabarbarum* reduced the *ALOX5* gene expression, a marginal or no effect was found for the root extracts originating from these plants. Moreover, both the *R. rhaponticum* and *R. rhabarbarum* extracts exerted a slight ability to inhibit the COX-2 and 5-LOX enzyme activities (the maximal inhibitory effect was about 20%) [35].

In this work, differences in the *R. officinale* petiole and root extract activity have been observed during analyses of pro-inflammatory cytokine secretion from PBMCs. The pro-inflammatory activity of various leukocyte populations and their cytokine-secretory activities are integral elements of cardiovascular diseases; therefore, an experimental model of human PBMCs was also applied in this study. Both the petiole and root extracts reduced the TNF-α and the IL-2 secretion from these cells. However, the IL-6 release from PBMCs was inhibited only by the petiole extract. This finding highlights the role of the flavonoid component in the petiole extract in suppressing the inflammatory response. Although the release of all the aforementioned cytokines is related to the activation of the NF-κB, they are, at least partly, regulated through different signaling pathways. While the p38 MAPK/NF-κB pathway is involved in the activation of TNF-α synthesis, the regulation of IL-6 synthesis is mediated by the gp130 protein [96]. Due to the higher presence of flavonoids in the *R. officinale* petiole extract compared to the root extract, they likely play a key role in regulating the synthesis and release of IL-6 during the inflammatory response. Inhibitory effects of flavonoids on different stages of either the activation of NF-κB itself or pro-inflammatory pathways triggered by its activation are well evidenced [97,98]. For instance, an inhibitory effect on the transcriptional activity of NF-κB and the JAK/STAT signaling was found for quercetin [99,100] and apigenin [101].

In general, current knowledge of the anti-inflammatory properties of rhubarb is mainly derived from traditional medicine, with only limited data obtained from scientific research on the biological activity of different types of extracts. Nevertheless, some evidence is available. Recent work of Lee et al. [102] includes a comparative study on antioxidant and anti-inflammatory activities of different organs of *R. rhabarbarum* and roots of *R. officinale*. Using an experimental model of RAW 264.7 macrophages, the authors found an anti-inflammatory effect of the examined rhubarb extracts. Similarly to our results, the authors observed a reduction in cell inflammatory response at a comparable range of concentrations (≤40 µg/mL). However, they employed a different methodology, specifically measurements of NO generation in LPS-stimulated macrophages. Additional information, including in vivo evidence, may be obtained from reports on the anti-inflammatory activity of individual compounds isolated from plant material. For instance, preparation of total free rhubarb anthraquinones from *R. officinale* was found to ameliorate severe acute pancreatitis in rats and reduce the level of pro-inflammatory markers, including the intestinal and serum levels of diamine oxidase (DAO), IL-1, IL-18, the high mobility group protein B1 (HMGB1), and lactate dehydrogenase (LDH). Analyses of molecular mechanisms of the observed anti-inflammatory action revealed a decrease in expressions of the toll-like receptor 4 (TLR-4), NF-κB, the apoptosis-associated speck-like protein containing a caspase recruitment domain (ASC), nucleotide-binding domain, leucine-rich-containing family, pyrin domain-containing-3 (NLRP3) inflammasome, caspase-1, and gasdermin D (GSDMD) genes [103]. In another study, emodin suppressed oxaliplatin-induced neuropathic pain by inhibiting COX2/NF-κB-mediated spinal inflammation in rats [104]. Furthermore, the COX-2 [105] and LOX-inhibitory activity of aloe-emodin was also reported. It has been demonstrated that aloe-emodin may act as a competitive inhibitor, reducing the LOX activity at micromolar concentrations (IC50 of 29.49 μM) [106].

The concentration range of the rhubarb extracts used in the present study is based on literature data and our earlier studies on other rhubarb species [35,36,37]. Literature evidence indicates that the physiologically achievable levels of most phytochemicals and their metabolites in blood plasma range from nanomoles to a few micromoles per liter. For instance, after dietary intake, hydroxycinnamic acids reached plasma concentrations of up to 1 µM, the isoflavone level was ranged from 0.46 to 4.04 µM, and the plasma concentrations of quercetin derivatives were 0.51–3.80 µM [107]. The concentration range of 1–50 μg/mL, used in our experiments on the anti-inflammatory properties of the examined extracts, corresponds to nano- and micromolar concentrations of their biologically active ingredients. To verify whether the observed anti-inflammatory effects of rhubarb extracts in HUVECs or PBMCs are not due to their cytotoxicity, the study design also included assessments of cellular safety for the examined plant extracts. In the aforementioned tests, the extract concentration range was extended up to 100 μg/mL to better recognize the effects of the extracts on cell viability and their cellular safety. In the 1–50 μg/mL concentration range, the extracts affected neither PBMCs membrane integrity nor viability. However, the treatment with higher concentrations (i.e., 75 and 100 μg/mL) induced a slight but statistically significant decrease in PBMCs’ metabolic activity and membrane integrity. In HUVECs, no effect of the examined extracts on cell viability was found in a full range of the used concentrations (1–100 μg/mL).

Our analyses of phytochemical composition and anti-inflammatory activity of *R. officinale* petiole and root extracts were complemented with comparative in vitro studies on their metabolism by gut microbiota. Once ingested, bioactive compounds derived from the plant undergo complex biotransformation processes within the human body, involving both intestinal and systemic mechanisms, which lead to the formation of diverse metabolites [108]. Therefore, the biological effects (including anti-inflammatory action) of natural products depend not only on the active compounds present in crude extracts but also on their metabolites formed after oral administration. Consequently, numerous methodological, substantive, and technical aspects regarding studies on the metabolism of plant-derived compounds have been widely discussed [109,110]. The increasing recognition of the significance of the intestinal microbiome in maintaining human physiological function and its involvement in the metabolism of xenobiotics, including chemical compounds present in plants [111], has prompted us to initiate studies on the microbiome effects on the chemical composition of the examined rhubarb extracts, reflecting the first stage of their metabolism. Our experiments provided the first comparison of the primary metabolites of *R. officinale* petioles and roots. Although their preliminary character, they form a basis for further, more complex analyses of metabolic transformation of the *R. officinale*-derived phytochemicals, both in terms of their structures and biological effects. Regarding the latter issue, future experiments would include studies of the activity of a mixture of postbiotic metabolites formed during gut microbiota-assisted digestion. Such an approach is currently not feasible when working with extracts or fractions obtained from plant material. A method for thoroughly purifying the post-incubation mixture, such as from bacteria-derived products, which would enable its addition to cell culture and the performance of ELISA tests on the obtained supernatants, has yet to be developed. Furthermore, in the case of an extract/fraction derived from plant material, both before and after incubation with gut microbiota, we are dealing with complex mixtures that cannot be reproduced using, for example, single chemical compounds produced by total synthesis. Nevertheless, our studies highlighted the main advantages and differences between the petiole and root extracts in the context of their future application. Of particular interest and promise is the observation that the majority of anthraquinones present in both extracts are metabolized into anthranoid derivatives during incubation with gut microbiota (Table 1). However, the extracts significantly differ in their profile, with petiole anthranoids metabolized primarily into monomeric compounds (e.g., emodin and chrysophanol derivatives), while root extracts produce dianthrone monoglycosides (sennidin A/C/D glucosides). This difference may significantly influence the biological effects of both extracts in humans, highlighting the need for further in vivo studies on both their metabolism and function.

## 5. Conclusions

In conclusion, our study provides the first comparative data on the phytochemical profile, cellular safety, and anti-inflammatory properties of both petiole and root extracts of *R. officinale*, along with analyses of their metabolization by the gut microbiota in vitro. While the root is traditionally used in medical applications, the petioles are generally overlooked and considered underutilized. Our combination of advanced phytochemical profiling with molecular biology and biochemical assays provided new insights into the bioactivity of both plant parts. In this sense, our research aligns with the zero-waste concept—making use of what was once considered waste. Surprisingly, the anti-inflammatory efficiency of the petiole extract was comparable, or even higher than that of the root extract.

The present work may be a background for researchers interested in anti-inflammatory properties of rhubarb and the interactions of the rhubarb-derived extracts with different blood components and the haemostatic system. However, further studies (in vivo works, in particular) are required to evaluate petioles as a new source of rhubarb active compounds and extracts, which might find their application in the industry, including the production of functional foods, dietary supplements, or even therapeutic agents.

## Figures and Tables

**Table 1:** UHPLC-DAD-MS^n^ data for metabolites from main native compounds from the petiole and root extracts of *R. officinale.*

Metabolite	Metabolite Identification	RT (min)	UV *λ*_max_ (nm)	[M–H]^−^ (*m*/*z*)	MS^2^ (*m*/*z*)	Source Compounds
**Petiole Extract**						
M1	(aloe)emodin	73.8	223	268.87	240.80	(aloe)emodin glycosides/ dianthrones
M2	hydroxy-emodin	62.2	221	285.05	240.78	emodin glycosides/ dianthrones
M3	acetyl-hydroxy-emodin	71.3	223	327.39	-	emodin glycosides/ dianthrones
M4	(aloe)emodin-physcion-dianthrone hexoside	72.0	222	685.30	253.79, 523.08	(aloe)emodin-physcion-dianthrone dihexosides
M5	chrysophanol isomer	11.5	190	253.26	209.81	physcion glycosides/ dianthrones
**Root extract**						
M6	rhein	71.0	222	282.96	238.74	rhein glycosides/ dianthrones
M7	acetyl-1,3,8-trihydroxy-6-methyl-9-oxanthranol/acetyl-1,3,8-trihydroxy-6-methyl-10-oxanthranol	66.0	220	313.15	268.75	emodin/chrysophanol glycosides/dianthrones
M8	sennidin A-8-*O*-glucoside	65.8	220	699.22	223.25, 537.07	sennoside A/ sennoside A esters
M9	sennidin C/D-8-*O*-glucoside/ sennidin C/D-8′-*O*-glucoside	64.8	220	685.21	223.75, 385.95, 479.05	sennoside C/D

RT, retention times. UV *λ*_max_, absorbance maxima in DAD spectra. [M–H]^−^, deprotonated molecules in MS spectra recorded in a negative mode. MS^2^, fragment ions in MS spectra recorded in a negative mode.

**Table 2:** Summary of the anti-inflammatory efficiency of the examined extracts from *R. officinale*. The table includes the maximum inhibitory effects found in HUVECs and PMBCs (for the extracts used at a concentration. of 50 μg/mL).

Parameters and Experimental Systems		Maximum Inhibitory Effect	
		Petiole Extract from *R. officinale*	Root Extract from *R. officinale*
Gene/protein expression and the enzyme activity tests	*COX2* (gene expression)/(HUVECs)	No effect	No effect
	COX-2 (enzyme activity)	63%	67%
	*ALOX5* (gene expression)/(HUVECs)	40%	22%
	5-LOX/(enzyme activity)	81%	78%
Cytokine release	TNF-α/(PBMCs)	73%	53%
	IL-2/(PBMCs)	44%	54%
	Il-6/(PBMCs)	78%	No effect

## Data Availability

The original contributions presented in this study are included in the article/Supplementary Materials. Further inquiries can be directed to the corresponding author.

## Supplementary Materials

The following supporting information can be downloaded at: https://www.mdpi.com/article/10.3390/nu17213455/s1. Table S1: Metabolites identified in butanol extract obtained from the petioles and roots of Rheum officinale; Figure S1: UHPLC-DAD-MS chromatograms of growth medium (BHI) and petioles extract from R. officinale after incubation with human gut microbiota (FS) after 0, 2, 5, 8, and 24 h of incubation; Figure S2: UHPLC-DAD-MS chromatograms of growth medium (BHI) and petioles extract from R. officinale after incubation with human gut microbiota (FS) after 0, 2, 5, 8, and 24 h of incubation; Figure S3: UHPLC-DAD-MS chromatograms of growth medium (BHI) and petioles extract from R. officinale after incubation with human gut microbiota (FS) after 0, 2, 5, 8, and 24 h of incubation; Figure S4: UHPLC-DAD-MS chromatograms of growth medium (BHI) and petioles extract from R. officinale after incubation with human gut microbiota (FS) after 0, 2, 5, 8, and 24 h of incubation.; Figure S5: UHPLC-DAD-MS chromatograms of growth medium (BHI) and petioles extract from R. officinale after incubation with human gut microbiota (FS) after 0, 2, 5, 8, and 24 h of incubation. Acquired using EIC 253.0 (−) mode. BHI+FS and BHI + extract, were presented as controls; Figure S6: UHPLC-DAD-MS chromatograms of growth medium (BHI) and roots extract from R. officinale after incubation with human gut microbiota (FS) after 0, 2, 5, 8, and 24 h of incubation; Figure S7: UHPLC-DAD-MS chromatograms of growth medium (BHI) and roots extract from R. officinale after incubation with human gut microbiota (FS) after 0, 2, 5, 8, and 24 h of incubation; Figure S8: UHPLC-DAD-MS chromatograms of growth medium (BHI) and roots extract from R. officinale after incubation with human gut microbiota (FS) after 0, 2, 5, 8, and 24 h of incubation; Figure S9: UHPLC-DAD-MS chromatograms of growth medium (BHI) and roots extract from R. officinale after incubation with human gut microbiota (FS) after 0, 2, 5, 8, and 24 h of incubation.. References [112,113] were cited in Supplementary Materials.
