# Supplementary material for: Phytochemical Profiling, Anti-Inflammatory Action, and Human Gut Microbiota-Assisted Digestion of Rheum officinale Petiole and Root Extracts—An In Vitro Study
Source: Nutrients. 2025 Nov 1;17(21):3455. doi: 10.3390/nu17213455 (PMC12609366; doi:10.3390/nu17213455)
Supplement: Supplementary file 1 [file nutrients-17-03455-s001.zip › 20.10_ Supplementary materials_R_officinale_MO.pdf]

SUPPLEMENTARY MATERIALS 1

Phytochemical profiling, anti-inflammatory action, and human gut microbiota-assisted digestion of *Rheum officinale* petiole and root extracts - an *in vitro* study

Oleksandra Liudvytska[1], Mariusz Kowalczyk[2], Justyna Krzyżanowska-Kowalczyk[2], Karolina Michaś[1], Maria Michalak[1], Aneta Balcerczyk[3], Weronika Skowrońska[4], Marcin Równicki[5], Agnieszka Bazylko[4], Monika A. Olszewska[6], Joanna Kolodziejczyk-Czepas[1*]

[1]Department of General Biochemistry, Faculty of Biology and Environmental Protection, University of Lodz, 90-236 Lodz, Poland

[2] Department of Biochemistry and Crop Quality, Institute of Soil Science and Plant Cultivation, State Research Institute, Czartoryskich 8, 24-100 Puławy, Poland

[3]Department of Oncobiology and Epigenetics, Faculty of Biology and Environmental Protection, University of Lodz, 90-236 Lodz, Poland

[4]Department of Pharmaceutical Biology, Faculty of Pharmacy, Medical University of Warsaw, Banacha 1, 02-097 Warsaw, Poland

[5]Microbiota Lab, Department of Pharmaceutical Microbiology and Bioanalysis, Medical University of Warsaw, 1 Banacha St., 02-097, Warsaw, Poland

[6]Department of Pharmacognosy, Faculty of Pharmacy, Medical University of Lodz, 1 Muszynskiego St., 90-151 Lodz, Poland

Correspondence to: J. Kolodziejczyk-Czepas, Department of General Biochemistry, Faculty of Biology and Environmental Protection, University of Lodz, Pomorska 141/143, 90-236 Lodz, Poland; email: joanna.kolodziejczyk@biol.uni.lodz.pl

Contents:

Table S1. Metabolites identified in butanol extract obtained from the petioles and roots of *Rheum officinale*.

Identifying detected compounds was mainly based on comparisons with reference spectra and interpretation of MS/MS spectra aided by SIRIUS and MetFrag [1]. Each tentatively identified metabolite was assigned an identification confidence level class, as suggested by [2]. The class 1 metabolites were confirmed using authentic reference standards, whereas class 4 compounds were only assigned elemental sum formula and had no identifiable structural features in their MS/MS spectra.

Figure S1. UHPLC-DAD-MS chromatograms of growth medium (BHI) and petioles extract from *R. officinale* after incubation with human gut microbiota (FS) after 0, 2, 5, 8, and 24 h of incubation. Acquired using EIC 267.0 (−) mode. BHI+FS and BHI + extract, were presented as controls. The marked place indicates the metabolite of emodin glycosides/dianthrones ((aloe)emodin, M1).

Figure S2. UHPLC-DAD-MS chromatograms of growth medium (BHI) and petioles extract from *R. officinale* after incubation with human gut microbiota (FS) after 0, 2, 5, 8, and 24 h of incubation. Acquired using EIC 285.0 (−) mode. BHI+FS and BHI + extract, were presented as controls. The marked place indicates the metabolite of emodin glycosides/dianthrones (hydroxy-emodin, M2).

Figure S3. UHPLC-DAD-MS chromatograms of growth medium (BHI) and petioles extract from *R. officinale* after incubation with human gut microbiota (FS) after 0, 2, 5, 8, and 24 h of incubation. Acquired using EIC 327.0 (−) mode. BHI+FS and BHI + extract, were presented as controls. The marked place indicates the metabolite of emodin glycosides/dianthrones (acetyl-hydroxy-emodin, M3).

Figure S4. UHPLC-DAD-MS chromatograms of growth medium (BHI) and petioles extract from *R. officinale* after incubation with human gut microbiota (FS) after 0, 2, 5, 8, and 24 h of incubation. Acquired using EIC 685.0 (−) mode. BHI+FS and BHI + extract, were presented as controls. The marked place indicates the metabolite of (aloe)emodin-physcion-dianthrone dihexosides ((aloe)emodin-physcion-dianthrone hexoside, M4).

Figure S5. UHPLC-DAD-MS chromatograms of growth medium (BHI) and petioles extract from *R. officinale* after incubation with human gut microbiota (FS) after 0, 2, 5, 8, and 24 h of incubation. Acquired using EIC 253.0 (−) mode. BHI+FS and BHI + extract, were presented as controls. The marked place indicates the metabolite of physcion glycosides/dianthrones (chrysophanol isomer, M5).

Figure S6. UHPLC-DAD-MS chromatograms of growth medium (BHI) and roots extract from *R. officinale* after incubation with human gut microbiota (FS) after 0, 2, 5, 8, and 24 h of incubation. Acquired using EIC 283.0 (−) mode. BHI+FS and BHI + extract, were presented as controls. The marked place indicates the metabolite of rhein glycosides/dianthrones (rhein, M6)

Figure S7. UHPLC-DAD-MS chromatograms of growth medium (BHI) and roots extract from *R. officinale* after incubation with human gut microbiota (FS) after 0, 2, 5, 8, and 24 h of incubation. Acquired using EIC 313.0 (−) mode. BHI+FS and BHI + extract, were presented as controls. The marked place indicates the metabolite of emodin/chrysophanol glycosides/dianthrones (acetyl-1,3,8-trihydroxy-6-methyl-9-oxanthranol/acetyl-1,3,8-trihydroxy-6-methyl-10-oxanthranol, M7).

Figure S8. UHPLC-DAD-MS chromatograms of growth medium (BHI) and roots extract from *R. officinale* after incubation with human gut microbiota (FS) after 0, 2, 5, 8, and 24 h of incubation. Acquired using EIC 699.0 (−) mode. BHI+FS and BHI + extract, were presented as controls. The marked place indicates the metabolite of sennoside A and its malonyl esters (sennidin A-8-*O*-monoglucoside, M8).

Figure S9. UHPLC-DAD-MS chromatograms of growth medium (BHI) and roots extract from *R. officinale* after incubation with human gut microbiota (FS) after 0, 2, 5, 8, and 24 h of incubation. Acquired using EIC 685.0 (−) mode. BHI+FS and BHI + extract, were presented as controls. The marked

place indicates the metabolite of sennosides C/D (sennidin C/D-8-*O*-monoglucose/sennidin C/D-8'-*O*-monoglucoside, M9).

Literature:

1. Ruttkies C, Schymanski EL, Wolf S, Hollender J, Neumann S. MetFrag relaunched: incorporating strategies beyond *in silico* fragmentation. J Cheminform. 2016; 8: 3. doi: 10.1186/s13321-016-0115-9.

2. Schrimpe-Rutledge AC, Codreanu SG, Sherrod SD, McLean JA. Untargeted Metabolomics Strategies-Challenges and Emerging Directions. J Am Soc Mass Spectrom. 2016; 27 (12): 1897-1905. doi: 10.1007/s13361-016-1469-y.

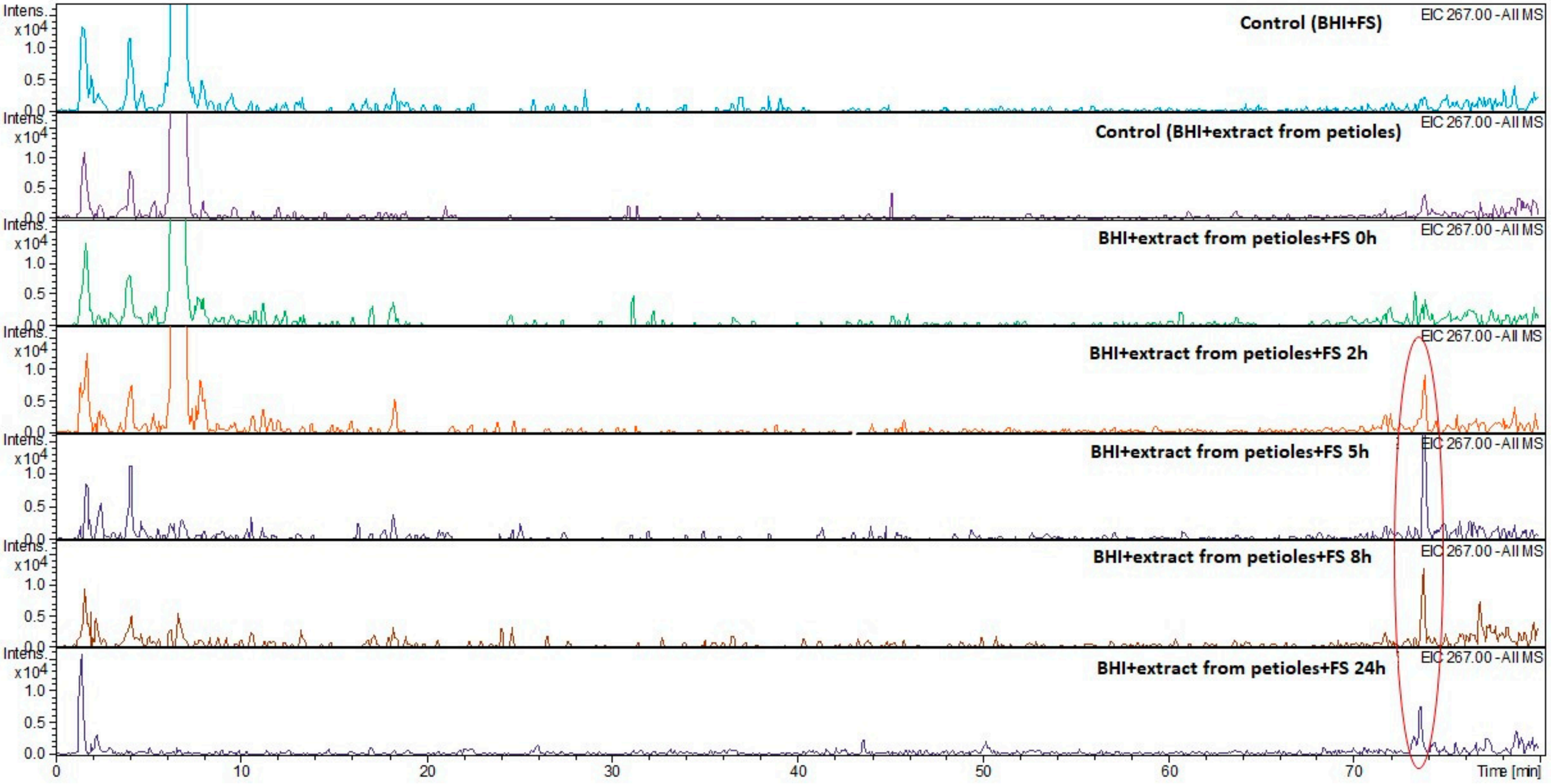

Figure S1. UHPLC-DAD-MS chromatograms of growth medium (BHI) and petioles extract from *R. officinale* after incubation with human gut microbiota (FS) after 0, 2, 5, 8, and 24 h of incubation. Acquired using EIC 267.0 (−) mode. BHI+FS and BHI + extract, were presented as controls. The marked place indicates the metabolite of emodin glycosides/dianthrones ((aloe)emodin, M1).

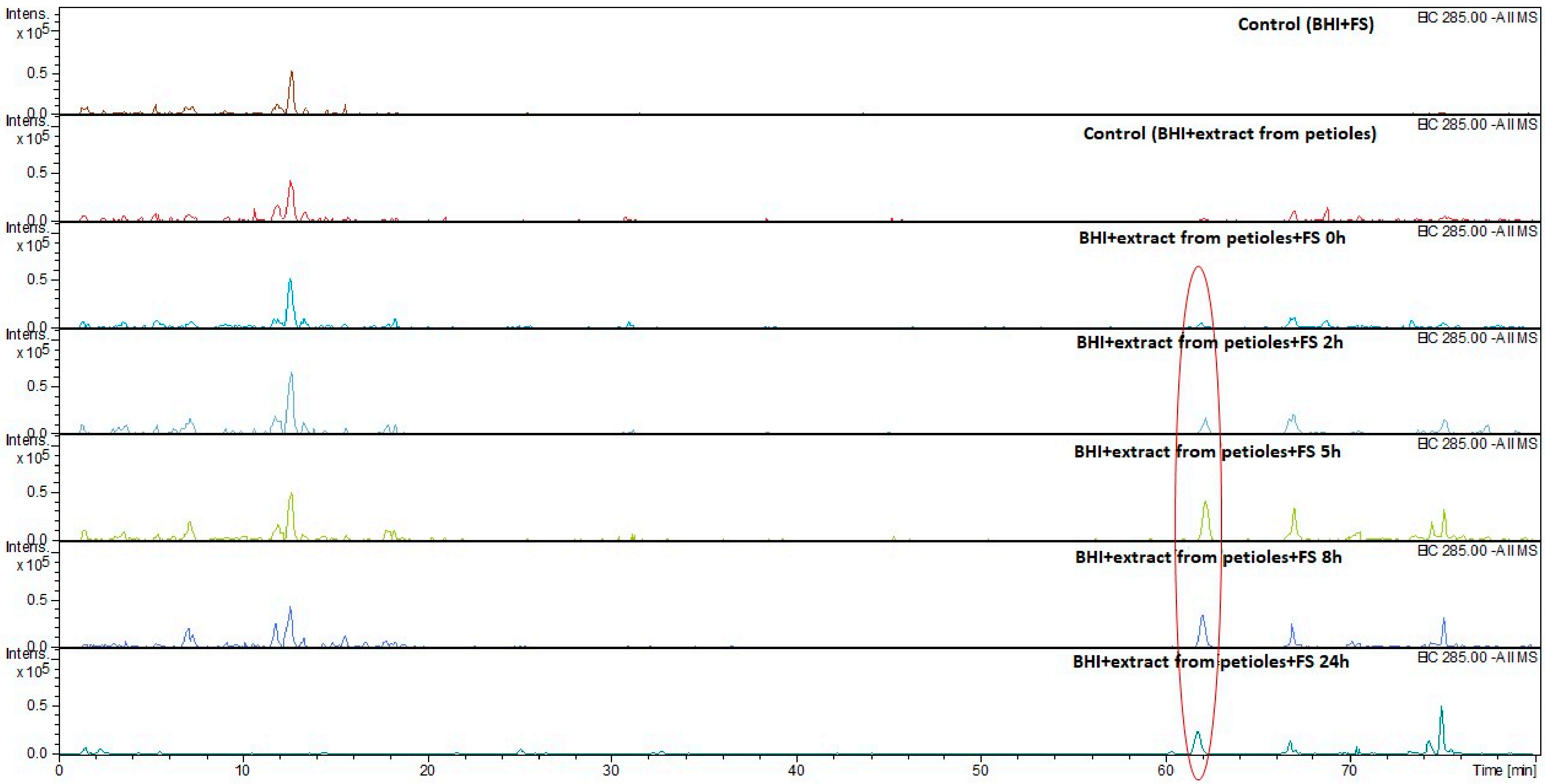

Figure S2. UHPLC-DAD-MS chromatograms of growth medium (BHI) and petioles extract from *R. officinale* after incubation with human gut microbiota (FS) after 0, 2, 5, 8, and 24 h of incubation. Acquired using EIC 285.0 (−) mode. BHI+FS and BHI + extract, were presented as controls. The marked place indicates the metabolite of emodin glycosides/dianthrones (hydroxy-emodin, M2).

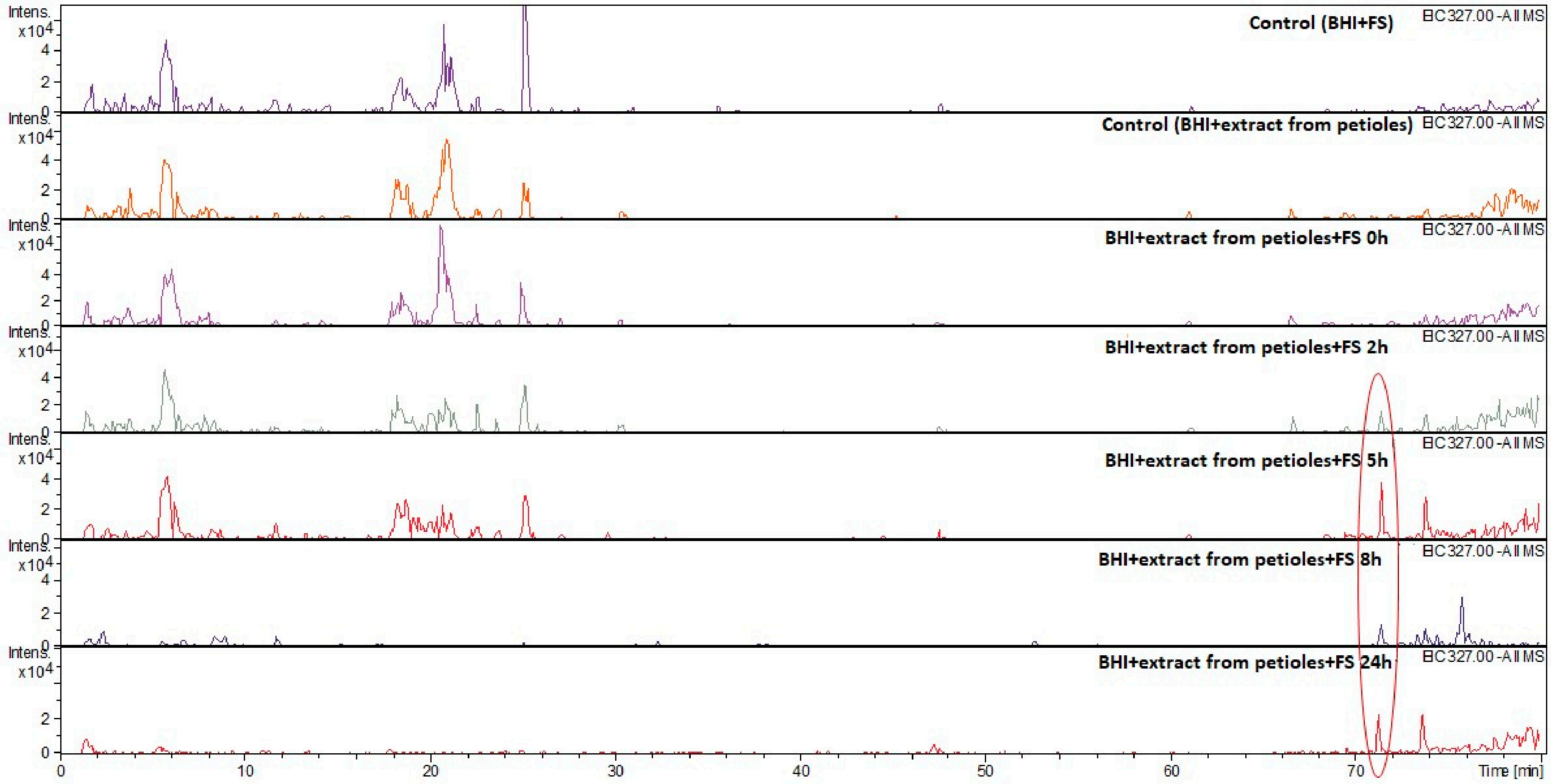

Figure S3. UHPLC-DAD-MS chromatograms of growth medium (BHI) and petioles extract from *R. officinale* after incubation with human gut microbiota (FS) after 0, 2, 5, 8, and 24 h of incubation. Acquired using EIC 327.0 (−) mode. BHI+FS and BHI + extract, were presented as controls. The marked place indicates the metabolite of emodin glycosides/dianthrones (acetyl-hydroxy-emodin, M3).

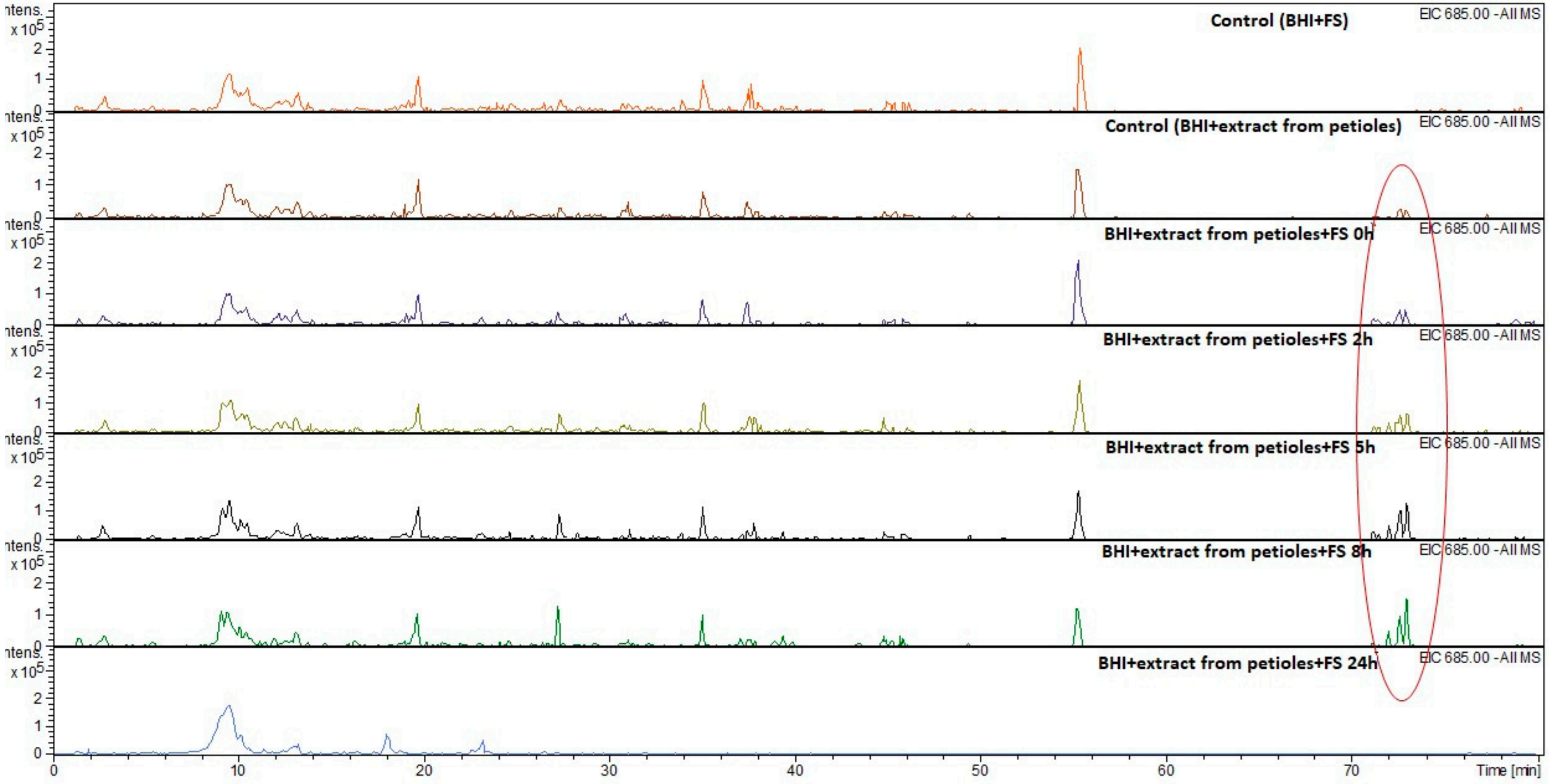

Figure S4. UHPLC-DAD-MS chromatograms of growth medium (BHI) and petioles extract from *R. officinale* after incubation with human gut microbiota (FS) after 0, 2, 5, 8, and 24 h of incubation. Acquired using EIC 685.0 (−) mode. BHI+FS and BHI + extract, were presented as controls. The marked place indicates the metabolite of (aloe)emodin-physcion-dianthrone dihexosides ((aloe)emodin-physcion-dianthrone hexoside, M4).

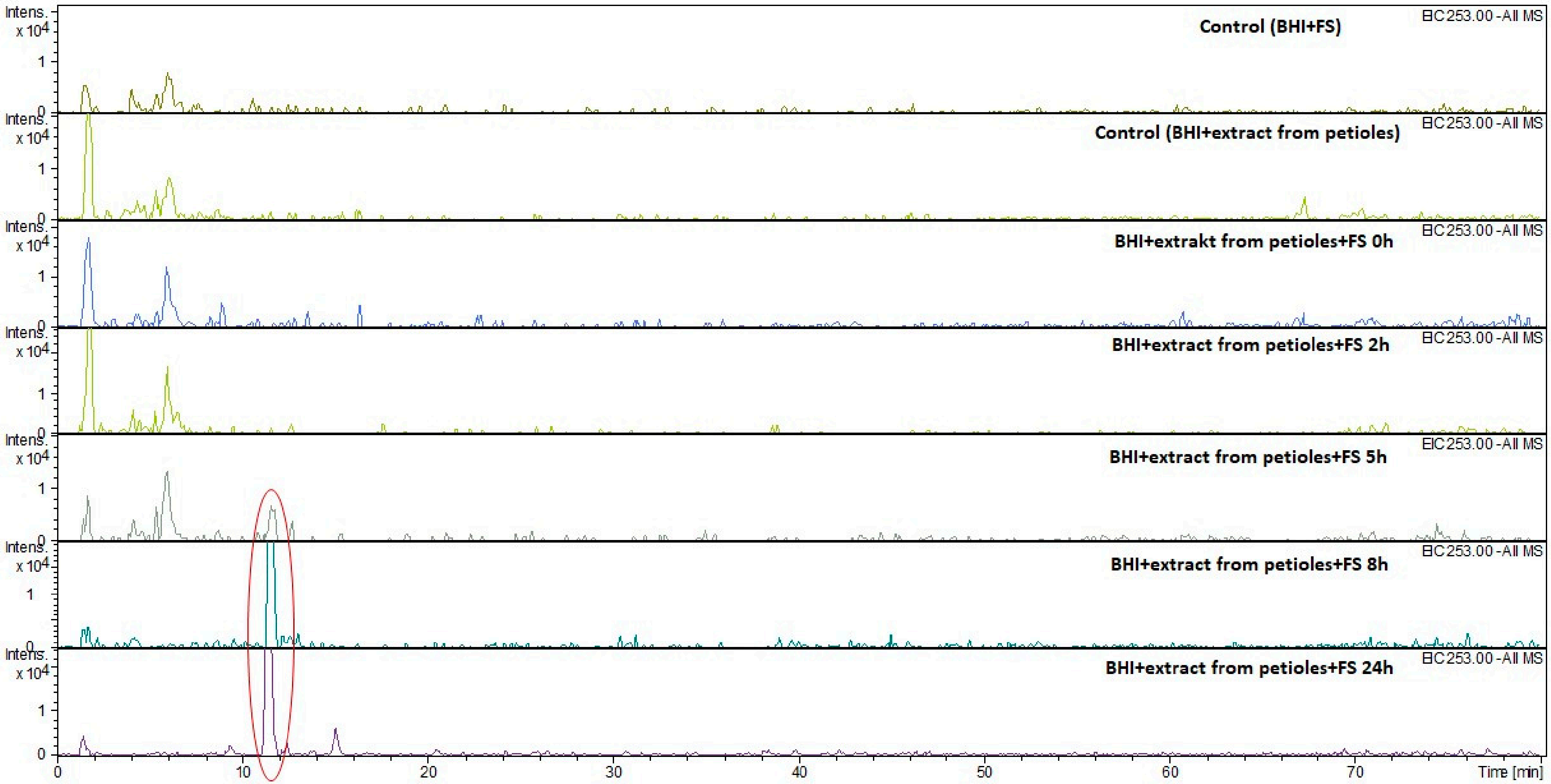

Figure S5. UHPLC-DAD-MS chromatograms of growth medium (BHI) and petioles extract from *R. officinale* after incubation with human gut microbiota (FS) after 0, 2, 5, 8, and 24 h of incubation. Acquired using EIC 253.0 (−) mode. BHI+FS and BHI + extract, were presented as controls. The marked place indicates the metabolite of physcion glycosides/dianthrones (chrysophanol isomer, M5).

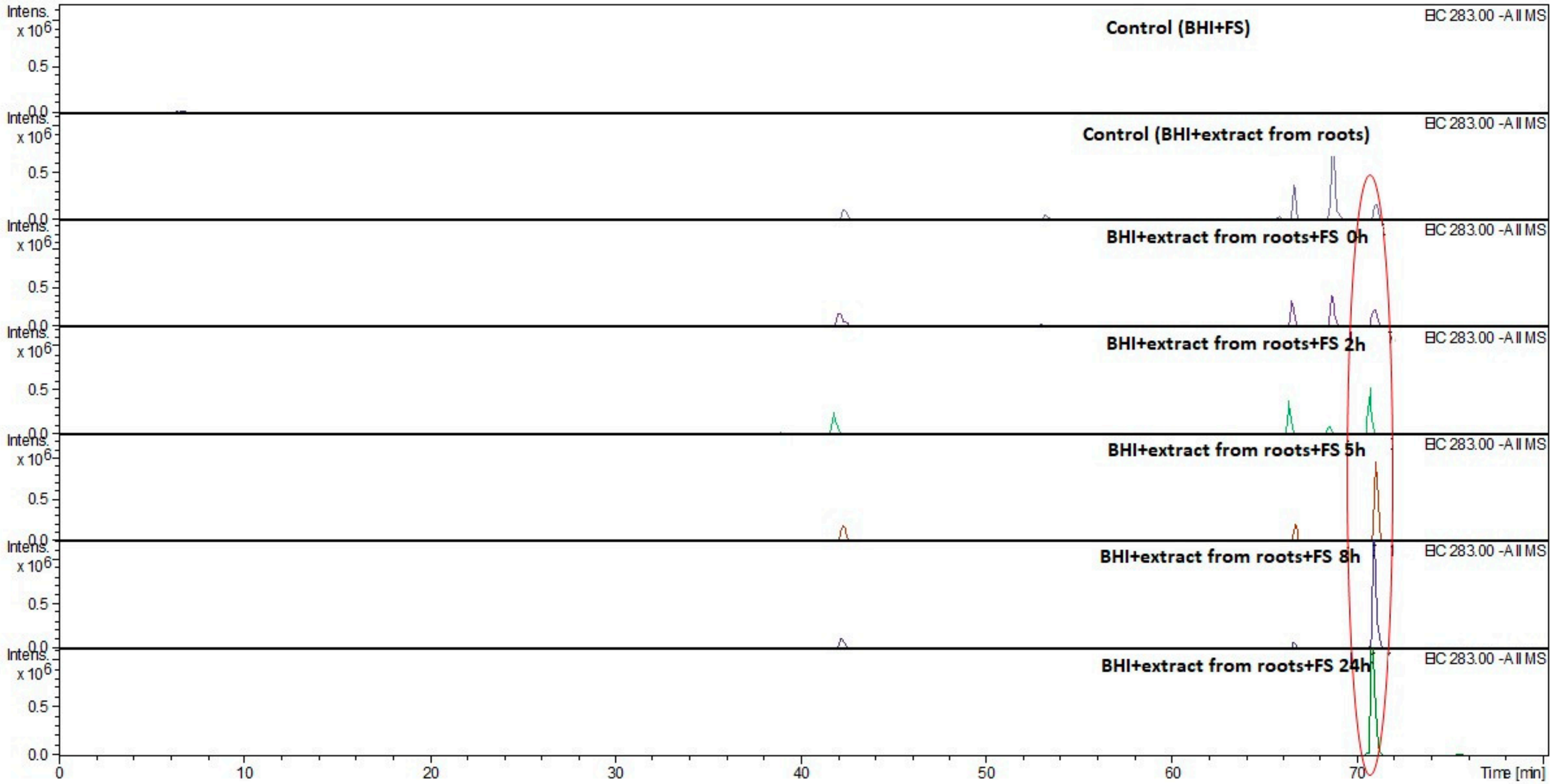

Figure S6. UHPLC-DAD-MS chromatograms of growth medium (BHI) and roots extract from *R. officinale* after incubation with human gut microbiota (FS) after 0, 2, 5, 8, and 24 h of incubation. Acquired using EIC 283.0 (−) mode. BHI+FS and BHI + extract, were presented as controls. The marked place indicates the metabolite of rhein glycosides/dianthrones (rhein, M6).

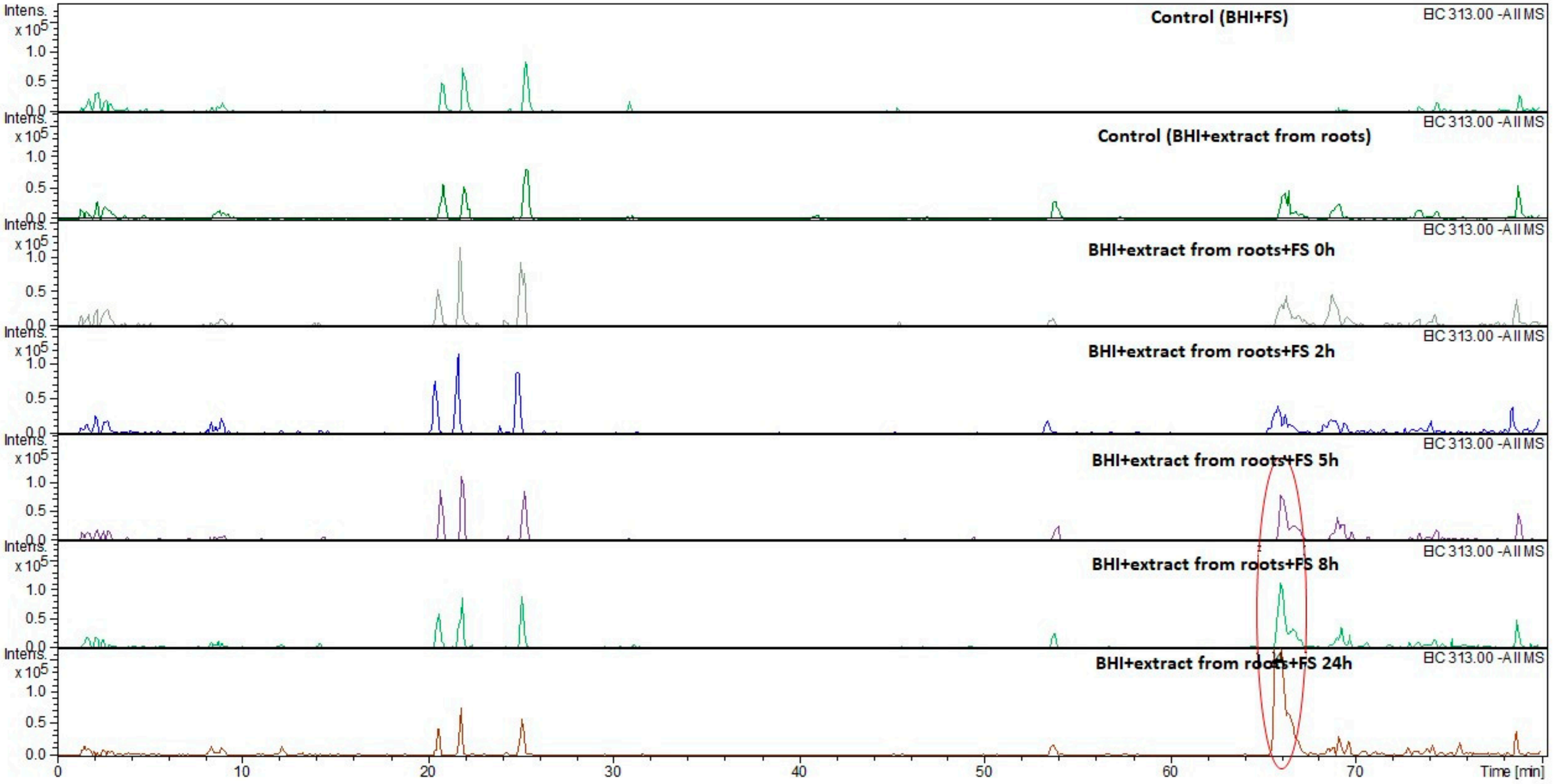

Figure S7. UHPLC-DAD-MS chromatograms of growth medium (BHI) and roots extract from *R. officinale* after incubation with human gut microbiota (FS) after 0, 2, 5, 8, and 24 h of incubation. Acquired using EIC 313.0 (−) mode. BHI+FS and BHI + extract, were presented as controls. The marked place indicates the metabolite of emodin/chrysophanol glycosides/dianthrones (acetyl-1,3,8-trihydroxy-6-methyl-9-oxanthranol/acetyl-1,3,8-trihydroxy-6-methyl-10-oxanthranol, M7).

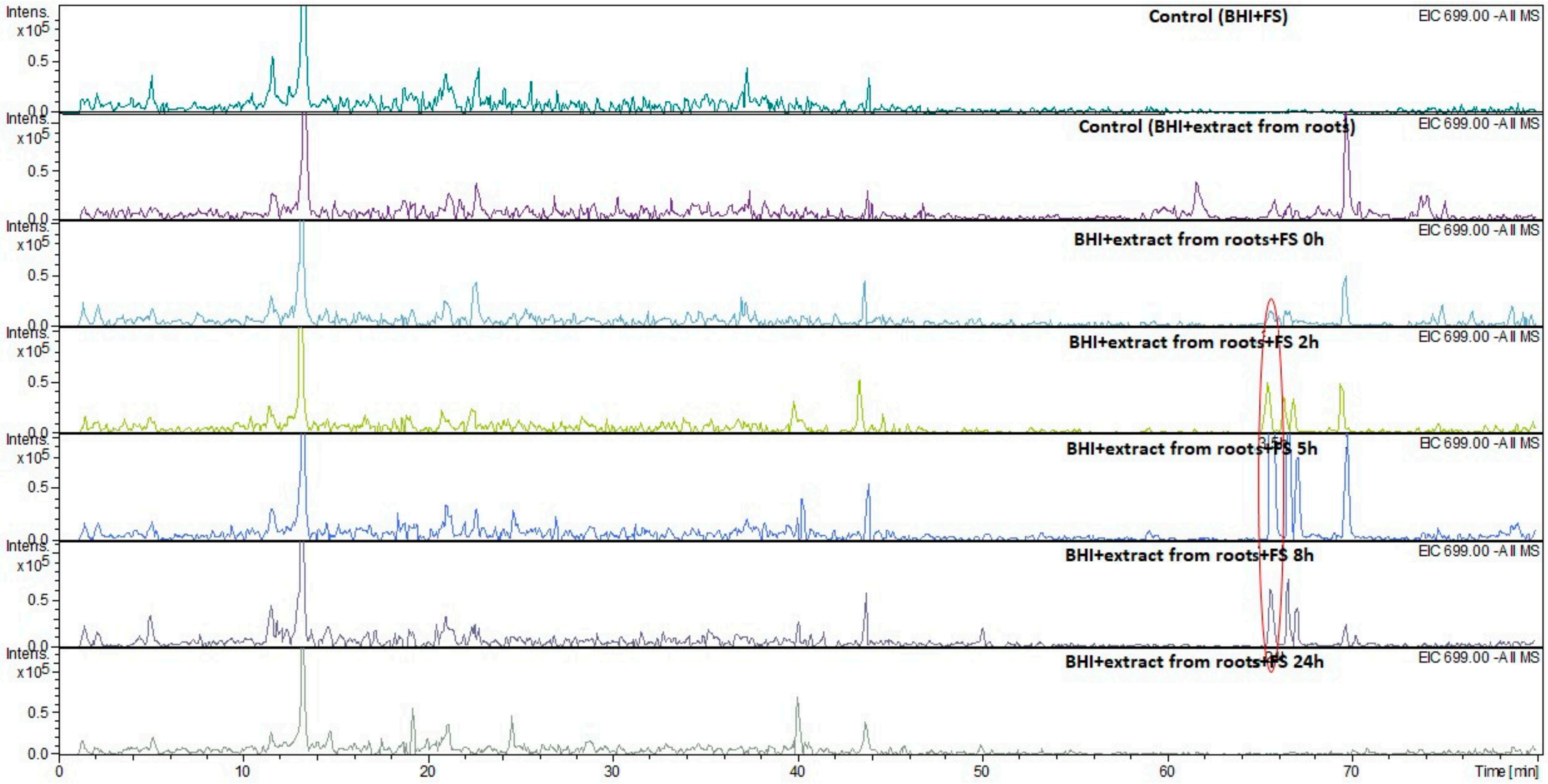

Figure S8. UHPLC-DAD-MS chromatograms of growth medium (BHI) and roots extract from *R. officinale* after incubation with human gut microbiota (FS) after 0, 2, 5, 8, and 24 h of incubation. Acquired using EIC 699.0 (−) mode. BHI+FS and BHI + extract, were presented as controls. The marked place indicates the metabolite of sennoside A and its malonyl esters (sennidin A-8-*O*-monoglucoside, M8).

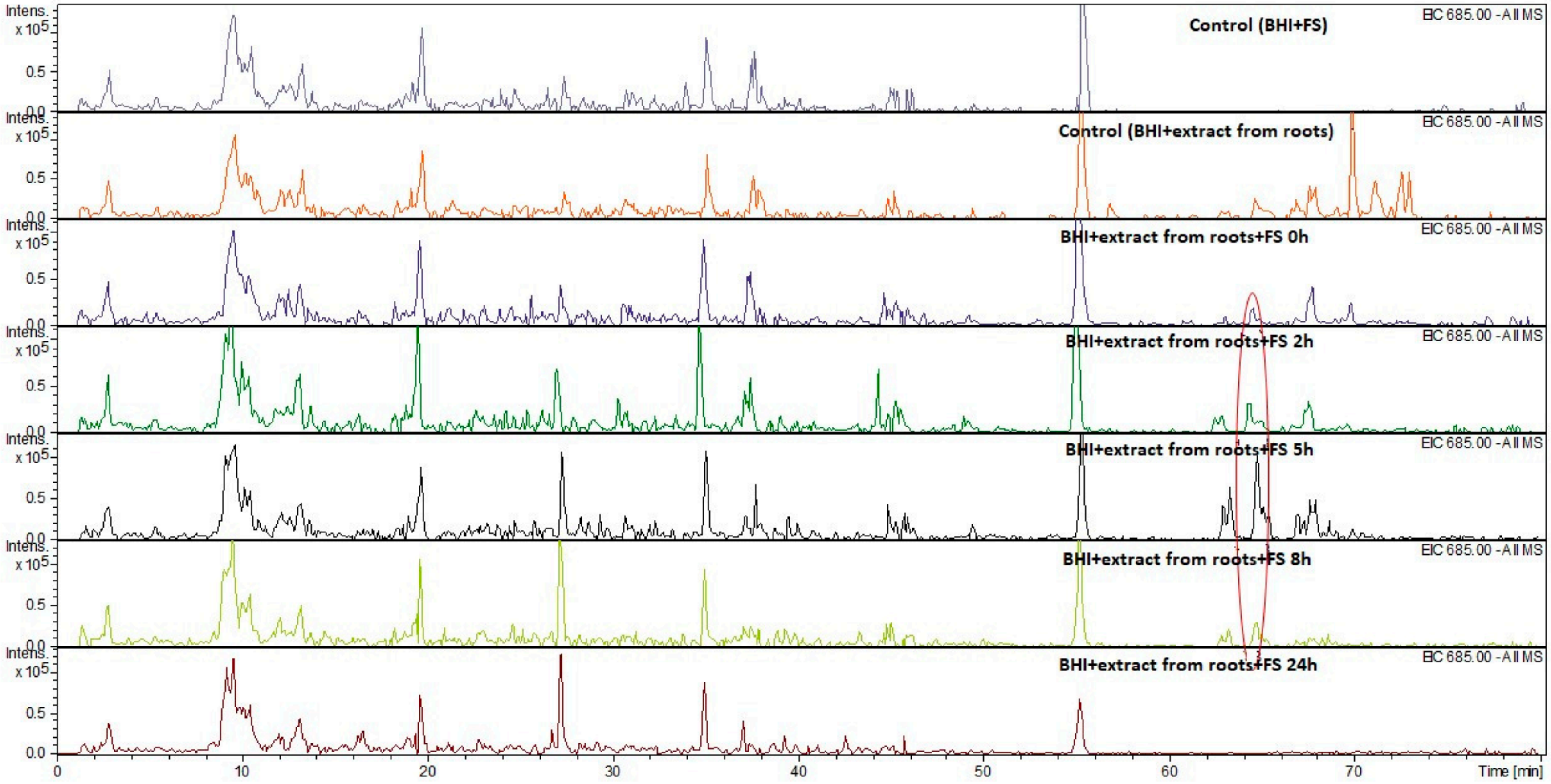

Figure S9. UHPLC-DAD-MS chromatograms of growth medium (BHI) and roots extract from *R. officinale* after incubation with human gut microbiota (FS) after 0, 2, 5, 8, and 24 h of incubation. Acquired using EIC 685.0 (−) mode. BHI+FS and BHI + extract, were presented as controls. The marked place indicates the metabolite of sennosides C/D (sennidin C/D-8-*O*-monoglucose/sennidin C/D-8’-*O*-monoglucoside, M9).
